# Genome-Wide Association Study of Anthracnose Resistance in Andean Beans (*Phaseolus vulgaris*)

**DOI:** 10.1371/journal.pone.0156391

**Published:** 2016-06-06

**Authors:** Grady H. Zuiderveen, Bilal A. Padder, Kelvin Kamfwa, Qijian Song, James D. Kelly

**Affiliations:** 1 Dept. of Plant, Soil and Microbial Sciences, Michigan State Univ., 1066 Bogue St., East Lansing, MI, 48824, United States of America; 2 USDA-ARS, 10300 Baltimore Ave., Soybean Genomics and Improvement Laboratory, BARC, Beltsville, MD, 20705–2350, United States of America; Università Politecnica delle Marche, ITALY

## Abstract

Anthracnose is a seed-borne disease of common bean (*Phaseolus vulgaris* L.) caused by the fungus *Colletotrichum lindemuthianum*, and the pathogen is cosmopolitan in distribution. The objectives of this study were to identify new sources of anthracnose resistance in a diverse panel of 230 Andean beans comprised of multiple seed types and market classes from the Americas, Africa, and Europe, and explore the genetic basis of this resistance using genome-wide association mapping analysis (GWAS). Twenty-eight of the 230 lines tested were resistant to six out of the eight races screened, but only one cultivar Uyole98 was resistant to all eight races (7, 39, 55, 65, 73, 109, 2047, and 3481) included in the study. Outputs from the GWAS indicated major quantitative trait loci (QTL) for resistance on chromosomes, Pv01, Pv02, and Pv04 and two minor QTL on Pv10 and Pv11. Candidate genes associated with the significant SNPs were detected on all five chromosomes. An independent QTL study was conducted to confirm the physical location of the Co-1 locus identified on Pv01 in an F_4:6_ recombinant inbred line (RIL) population. Resistance was determined to be conditioned by the single dominant gene *Co-1* that mapped between 50.16 and 50.30 Mb on Pv01, and an InDel marker (NDSU_IND_1_50.2219) tightly linked to the gene was developed. The information reported will provide breeders with new and diverse sources of resistance and genomic regions to target in the development of anthracnose resistance in Andean beans.

## Introduction

The common bean (*Phaseolus vulgaris* L.) is the most important grain legume grown globally for direct human consumption and is particularly important in many developing countries [1]. Historically, and still today, the common bean is grown and consumed in developing countries in Africa, Latin America, and Asia with eight of the top ten producing countries of dry beans considered as developing [2]. In the tropics and subtropics, bean yields are greatly reduced as beans are susceptible to numerous diseases caused by fungal pathogens [3]. Anthracnose, caused by the fungus *Colletotrichum lindemuthianum*, is one of the most economically important diseases of common bean [4], and can cause devastation to farmers’ fields, resulting in yield losses as high as 95% in susceptible cultivars [5]. Being a seed borne pathogen makes anthracnose especially problematic for small scale farmers from many developing countries as they save their own seed from year to year [6]. Transmission of anthracnose through the seed is very efficient [7], which makes clean seed an essential component of anthracnose prevention around the world. Effective clean seed programs have resulted in diminished cases of anthracnose in America and Europe [8]. However, clean seed is not a viable option for many farmers in Latin America and Africa as these seed programs require organized seed production in areas where the pathogen is not endemic [6]. Thus, infected seeds of susceptible varieties still serve as a main source of disease inoculum in subsistence farming systems [4].

**Fig 1:**
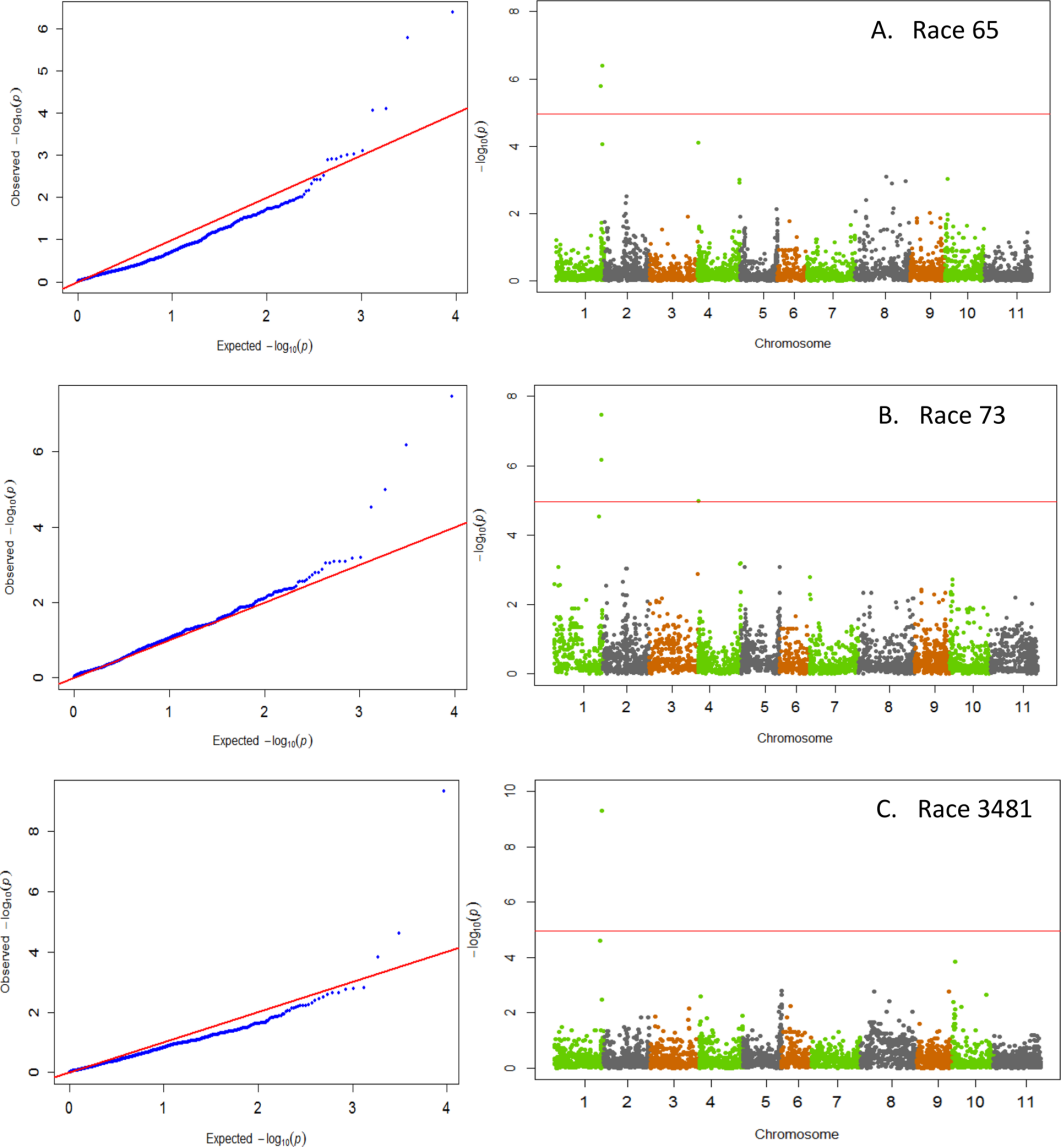
QQ and Manhattan Plots showing candidate SNPs and P-values from GWAS using MLM for anthracnose resistance. From top to bottom include results for anthracnose resistance to A) race 65, B) race 73, and C) race 3481 on Pv01. Red line on Manhattan Plots is the significance threshold of P = 1.03 x 10^−5^ after Bonferonni correction of α = 0.05.

Incorporating genetic resistance to anthracnose is the area of research and development that holds the most promise for reducing the effects of the pathogen in common bean. To date, over 100 pathogenic races have been reported globally using the 12 differential cultivars and the binary naming system for race identification [6, 7]. Seventeen independent loci, *Co-1* to *Co-17*, conditioning resistance have been mapped to the eight chromosomes Pv01, Pv02, Pv03, Pv04, Pv07, Pv08, Pv09 and Pv11 in addition to seven other genes *Co-u*, *Co-w*, *Co-x*, *Co-y*, *Co-z*, *CoPv02c* and *CoPv09c*, some of which have been mapped to the same chromosomes where the numbered loci are located [6, 9, 10]. A graphical depiction of the mapped *Co*-genes is shown in Fig 1 in the publication by Meziadi et al. [11]. Anthracnose resistance is dominant at all loci except the co-8 locus, and multiple alleles have been identified at the Co-1, Co-3, Co-4, and Co-5 loci. More recently, resistance genes *Co-15* and *Co-16* in the Brazilian landraces Corinthiano [12] and Crioulo 159 [13, 14], respectively have been mapped to Pv04. The *Co-13* gene in the landrace Jalo Listras Pretas from Brazil and *Co-17* in SEL1308 were recently mapped to Pv03 [15,16]. In addition, the co-localization of the major *Co-1* gene (*Co-1*^*4*^ allele) with the *Phg-1* gene conditioning resistance to angular leaf spot was confirmed on Pv01 [11, 17]. Co-segregation of the *Co-10* gene with the *Phg-ON* (renamed *Phg-3*) for angular leaf spot in the black bean cultivar Ouro Negro was also confirmed on Pv04 [18]. A major resistance cluster consisting of the Co-3 locus with five alleles, one formerly known as *Co-9* (renamed *Co-3*^*3*^), *Co-10* (renamed *Co-3*^*4*^), *Co-7* (renamed *Co-3*^*5*^) [19], and the recently proposed genes *Co-15* and *Co-16*, are all located on Pv04 along with *Ur-5*, *Ur-14*, and *Phg-3* genes for rust and angular leaf spot [20]. The *Co-17* is the second anthracnose resistance gene (*Co-13*) to be mapped to Pv03, and a new resistance locus described in cultivar Xana could be the previously described *Co-u* gene on Pv02 [10].

In addition to the qualitative resistance reported, quantitative resistance loci (QRL) conditioning resistance to 3 races of *C*. *lindemuthianum* have been mapped in Brazilian carioca cultivar ICA-UNA and two major effect QRLs coincided with two previously characterized major genes *Co-u* and *Co-5* located on Pv02 and Pv07, respectively [21]. Additional QRL were identified on Pv01, Pv03, Pv04, Pv05, Pv07, Pv08 and Pv09 in a QTL study of resistance to races 23 and 1545 in nuna bean PHA1037 from Spain [22]. The only unique resistance locus detected resided on Pv05. Having access to the whole-genome sequence of *Phaseolus* [23], has resulted in the fine mapping of many of these QRLs and resistance sources including: *Co-x* [24], *Co-1*^*2*^ [25] and the *Co-4*^*2*^ [26] and the discovery of new genomic regions and candidate genes associated with anthracnose resistance [22]. The *Co-x* gene was fine mapped to Pv01, independent of the Co-1 locus, and to a syntenic region, located at one end of soybean (*Glycine max*) chromosome 18 that carries *Rhg1*, a major gene conditioning resistance to soybean cyst nematode [24]. Fine mapping of the Co-4 (COK-4) locus on Pv08 revealed 18 copies of the COK-4 gene in a 325kb segment of that chromosome [26]. Given the current information on resistance sources many mapped and tagged with molecular markers, bean breeders are poised to build more selective gene pyramids to stem the rapid evolution of new races of the pathogen. Finding new resistance sources in Andean beans would provide an additional reservoir of materials within this gene pool, and offer the possibility of pyramiding anthracnose resistance genes from both gene pools, which should result in more durable resistance.

The objective of this study was to utilize a subset of 226 lines from the Andean Diversity Panel (ADP) to screen against eight races of anthracnose to identify new sources of anthracnose resistance in Andean beans, and explore the genetic basis of the resistance using GWAS to identify and fine map new genomic regions controlling resistance.

## Materials and Methods

A subset of 226 bean lines selected from the ADP [27] and four checks, was screened with eight different races of anthracnose in the greenhouse during spring and fall of 2014. Many of the original 396 lines included in the ADP [27] come from breeding programs in the U.S. and from African countries and South American countries where Andean beans originated (S1 Table). The subset of the ADP that was selected was based on ability to produce seed under Michigan growing conditions. The anthracnose races used in the study are named based on the standardized system of virulence of each race to the 12 differential bean cultivars [4]. The choice of races was made to include common cosmopolitan races, highly virulent Andean races, and highly virulent races across both gene pools (S3 Table). Race 7 is an Andean race that has been found in South America, and is also found in high frequency in the United States [28, 29]. Races 65 and 73 are both common Mesoamerican races in North, Central, and South America, with race 73 representing over 25% of all isolates identified in an anthracnose diversity study [28]. Race 39 is an Andean race that is known for being virulent on numerous Andean differential cultivars including Kaboon, Perry Marrow, and Michigan Dark Red Kidney (MDRK). Race 55 possess the same virulence pattern as race 39, but is also virulent on the Andean differential Widusa, resulting in susceptibility of all the differentials known to possess an allele of the Co-1 locus [21]. Race 109 was included as it also is virulent on the Andean differential cultivars Kaboon and Perry Marrow. Lastly, highly virulent races 2047 and 3481 were included in the study as they are virulent across both gene pools (see Table 9.1 [6]), and result in susceptibility of nearly the entire differential series. Race 2047 is virulent on 11 of the 12 differentials including all the Andean differential cultivars, whereas race 3481 is highly virulent on 7 of the 12 differentials including G2333 the most resistant genotype in the differential series.

For each bean line included in the study, six seedlings were grown in trays containing standard potting soil in the Michigan State University greenhouses, East Lansing, MI. Inoculations were done by spraying a suspension of 1.2 x 10^6^ *C*. *lindemuthianum* conidia ml^-1^ onto the leaves and stems of seedling plants. Plants were then maintained under high humidity (>80%) in a mist chamber for a minimum of three days. Symptoms of anthracnose were observed on susceptible plants 8–10 days after initial inoculation and rated a 0–5 scale [30]. Ratings were assessed as follows: 0, no symptoms observed; 1, pinpoint lesions present on stem and hypocotyl; 2, small surface lesions on stem and leaf veins; 3, large, sunken lesions present on stem; 4, lesions sunken to the center of the stem, wilting of chlorotic leaves; 5, plant killed by pathogen. Some ADP lines were heterogeneous mixtures for reaction to specific anthracnose races and were removed from the final analysis, and that data is reported in S1 Table. Correlations between races that clustered on the same region of a given chromosome based on GWAS was run using the following equation: $r_{X,Y}=\frac{{COV}_{X,Y}}{\sqrt{V_{X}V_{Y}}}$

Where: *X*, *Y* are the anthracnose disease rating of the clustered races, *r*_*X*,*Y*_ is the correlation coefficient, *COV*_*X*,*Y*_ is the covariance between the two clustered anthracnose races, and $\sqrt{V_{X}V_{Y}}$ is used to scale the covariance to vary between -1 and 1.

DNA was collected from young leaf tissues of ADP genotypes grown in the greenhouse at Michigan State University using a modified CTAB (Hexadecyltrimethyl ammonium bromide) extraction protocol [31]. The DNA concentrations were measured using a Nanodrop spectrophotometer, and its quality was checked on an agarose gel. The Andean panel was previously genotyped using an Illumina BARCBean6K_3 BeadChip with 5398 SNPs [32].

The population structure in the ADP was determined using principal component analysis (PCA) implemented in EIGENSTRAT [33] as described in [34]. After filtering for low quality and monomorphic SNPs as well as for minor allele frequency (MAF>0.02), a total of 4850 SNPs were retained for the PCA and association analysis. The kinship matrix developed using identical by descent method implemented in TASSEL was included in the association analysis to correct for cryptic relatedness. A Mixed Linear Model (MLM) [35] was run in TASSEL to determine the SNP-trait associations. The MLM equation used in the analysis was as follows:

Y=Xα+Pβ+Kμ+ε

Where: Y is the phenotype of a genotype; X is the fixed effect of the SNP; P is the fixed effect of the population structure; K is the random effect of the relative kindship; ε is the error term and is assumed to be normally distributed with a mean of zero. The conservative Bonferonni corrected p = 1.0 x 10^−5^ (for α = 0.05 and 4850 SNPs) was used to determine the significance threshold for SNPs.

The common bean genome [23] was browsed using Jbrowse on Phytozome v10 [36] to identify positional candidate genes associated with the significant SNPs. The functional annotation for the gene was then identified on Phytozome v10 in order to infer the possible role of the gene in conferring anthracnose resistance.

Resistance to anthracnose was also investigated in an F_4:6_ Middle American black bean RIL population developed from a cross between Jaguar known to possess the *Co-1* gene with resistance to race 73 [37] and Puebla 152 (landrace cultivar known to be susceptible to race 73). The RIL population, along with the parents, was genotyped using an Illumina BARCBean6K_3 BeadChip with 5398 SNPs [32]. The SNP-based genetic map was developed using JoinMap 4 [38]. The SNP genotyping data for the population were manually inspected in Excel, and SNPs with no calls, those that were monomorphic between parents, and any in which the parents were heterozygous were eliminated. The markers were ordered on the map and the genetic distances between the markers were determined using the regression mapping algorithm and Kosambi’s mapping function. The LOD range was between a minimum of 2 and a maximum of 10, with the remaining parameters left at JoinMap defaults for linkage analysis. Win QTL Cartographer V2.5_011 [39] was utilized to conduct the QTL analysis. The Linkage map was drawn using Mapchart 2.3 for Windows [40].

InDel markers developed under Common Bean Coordinated Agricultural Project (BeanCAP) [41] (www.beancap.org) were used to screen for polymorphism using bulk segregant analysis and the Jaguar and Puebla 152 parents of the RIL population. Fifty InDel markers located on Pv01 from 1.4 to 51.25 Mb were initially used but with the discovery of a significant SNP ss715645252 at 50.22 Mb in the QTL study, research was later focused on InDels located from 49.5 to 50.95 Mb. PCR was performed using thermocycler PTC 100 in 0.2ml PCR tubes containing 1x Go Taq buffer (Promega, Madison, WI), dNTPs (0.5mM), primers forward and reverse (0.25μM), 1U Go Taq and 50ƞg of template DNA. The amplification was carried out using PCR program by first denaturing tubes at 95°C for 3 min., then 45 cycles at 95°C for 20 sec, 55°C for 30 sec, 72°C for 1 min and final extension at 72°C for 10 min. PCR products were resolved on 3% agarose gel containing ethidium bromide, run in 1x TAE buffer and visualized in gel documentation system (Bio-Rad Labs, Hercules, CA). Co-segregation between a single polymorphic InDel marker located at 50.22 Mb was conducted against 97 RILs screened against anthracnose race 73. Further validation of the same marker was conducted on 30 bean genotypes from both the Andean and Mesoamerican gene pools that either possessed the *Co-1* gene or an allele or not.

## Results and Discussion

Developing common bean cultivars with resistance to anthracnose is one of the most effective ways of controlling this important disease. The identification of sources of resistance as well as understanding the underlying genetic basis of anthracnose resistance is critical to this effort. In the current study, resistance to eight races of *C*. *lindemuthianum* as well as the genetic basis of the resistance was investigated in a diverse group of Andean bean lines. Resistant lines were identified within the 226 Andean bean lines but few were resistant to all eight races (7, 39, 55, 65, 73, 109, 2047, and 3481) of *C*. *lindemuthianum* included in the study (S1, S2 and S3 Tables). In general, the Mesoamerican or mixed races (65, 73, and 3481), not widely pathogenic on Andean beans, expressed the lowest level of virulence on members of the ADP. Race 3481, despite being a highly virulent race, was virulent on only 28.8% of the ADP, while races 65 and 73 were virulent on 36.7% and 34.5% of the panel, respectively. It would appear that the resistance within the ADP to these three races can be attributed to the Co-1 locus and its alleles, as all three races were not virulent on the *Co-1* (MDRK), the *Co-1*^*3*^ (Perry Marrow), or *Co-1*^*2*^ (Kaboon) alleles at the Co-1 locus. These data indicate the potential value of resistance genes in ADP to breeders working to improve Mesoamerican genotypes susceptible to highly virulent Mesoamerican races of *C*. *lindemuthianum*.

Conversely, the other 5 races of *C*. *lindemuthianum* were virulent on more than 50% of the cultivars screened. Resistance to virulent Andean races 39 and 55 was 41.6% and 41.2%, respectively in the panel. Both races 39 and 55 were virulent on the majority of alleles at the Co-1 locus, and thus it can be assumed that resistance observed is provided by a locus less common within Andean beans. Resistance to races 7 and 109 were less prevalent than races 39 and 55 with only 37.6% of the lines resistant to race 7 and 34.5% of the ADP resistant to race 109. The most virulent race on the ADP was race 2047, in which only 4.4% of the lines exhibited some level of resistance. This was not unexpected as race 2047 defeats all Andean genes in the differential series [6].

Within the ADP, certain lines possessed resistance to multiple races of *C*. *lindemuthianum*. Among the 226 lines, 28 were resistant to six or more of the eight races included in the study (S2 Table). Nineteen of these lines were bred in North America. The cultivar Red Hawk was one of the 28 lines resistant to at least six races of anthracnose, and illustrates the potential value of gene pyramiding as it is known to possess the Andean resistance gene *Co-1* and the Mesoamerican resistance gene *Co-2* [42]. Only one line Uyole 98, a yellow bean variety from Tanzania, was resistant to all eight races of anthracnose. Previous work had identified Uyole 98 as having resistance to another fungal pathogen, *Pseudocercospora griseola*, the causal agent of angular leaf spot [43]. Identifying resistance in a diverse group of bean genotypes differing in seed and adaptation traits will provide breeders with valuable parental materials for use in local breeding programs.

**Fig 2:**
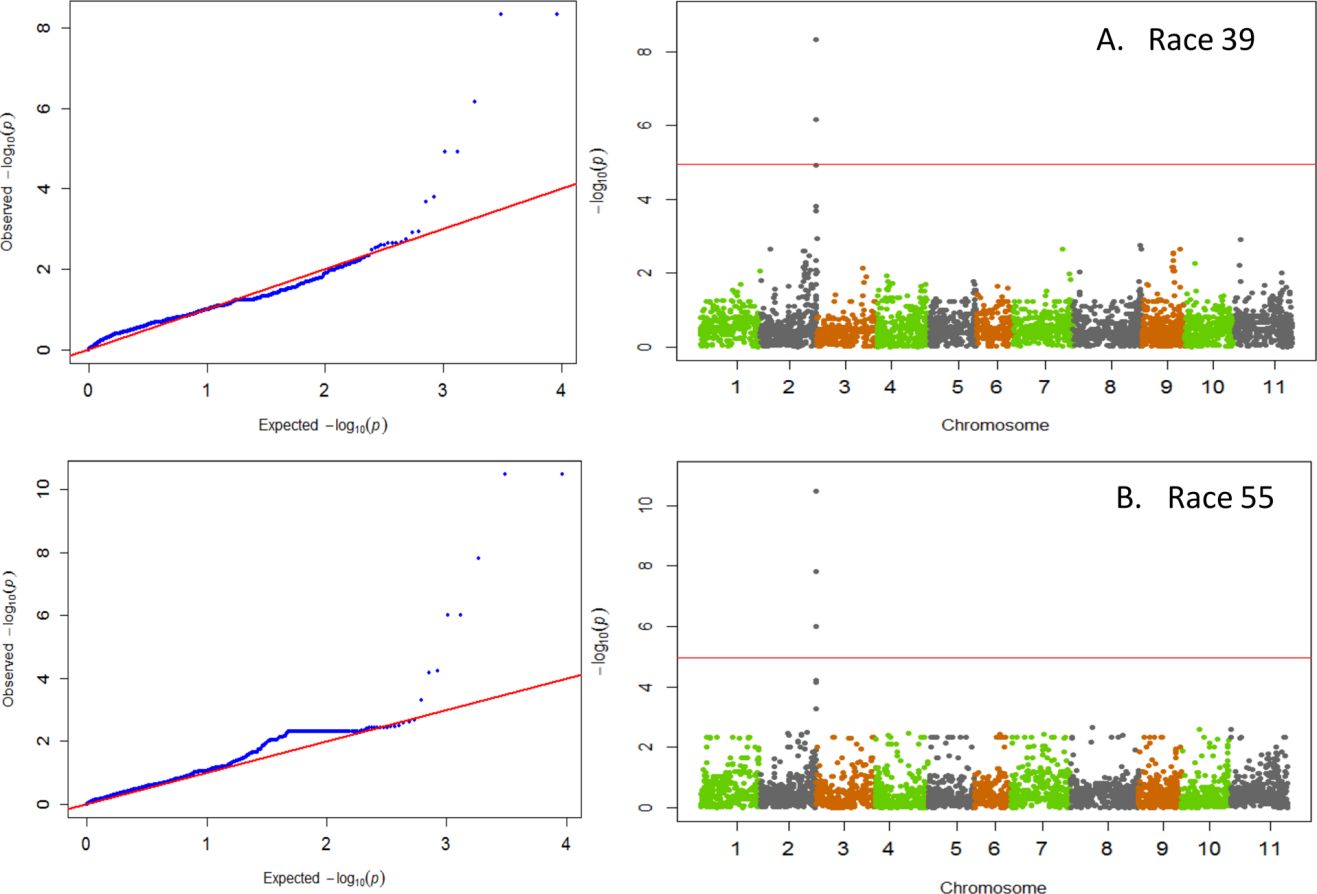
QQ and Manhattan Plots showing candidate SNPs and P-values from GWAS using MLM for anthracnose resistance. From top to bottom include results for anthracnose resistance to A) race 39 and B) race 55 on Pv02. Red line on Manhattan Plots is the significance threshold of P = 1.03 x 10^−5^ after Bonferonni correction of α = 0.05.

**Fig 3:**
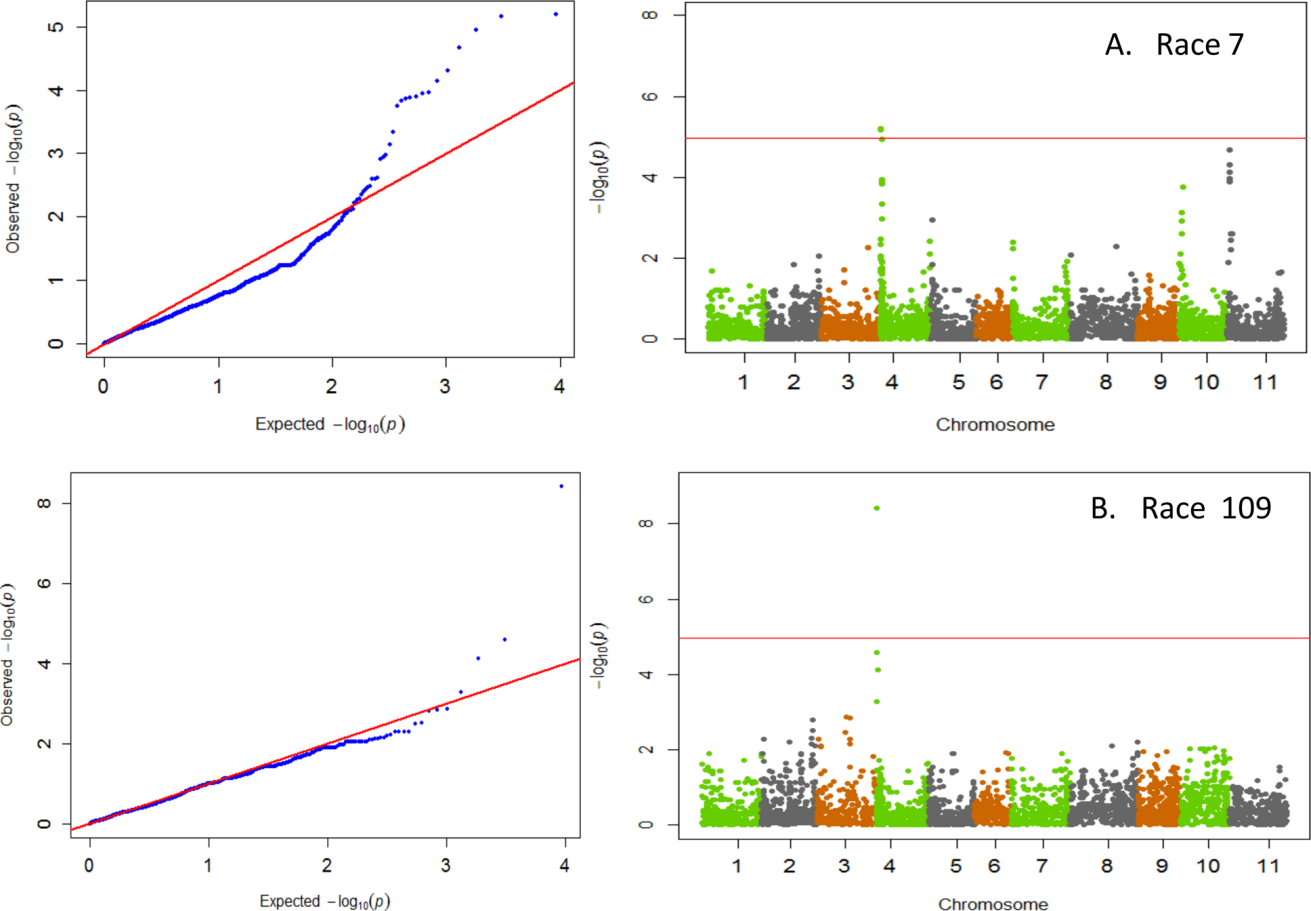
QQ and Manhattan Plots showing candidate SNPs and P-values from GWAS using MLM for anthracnose resistance. From top to bottom include results for anthracnose resistance to A) race 7 and B) race 109 on Pv04, with minor resistance loci on Pv10 and Pv11 for race 7. Red line on Manhattan Plots is the significance threshold of P = 1.03 x 10^−5^ after Bonferonni correction of α = 0.05.

GWAS results indicated that significant major QTL for resistance within Andean beans resided on three chromosomes, Pv01, Pv02, and Pv04 (Table 1). Races 65, 73, and 3481 all identified resistance on Pv01 (Fig 1). The resistance within the ADP to these three races showed a strong correlation expected from resistance mapping to the same region with correlation coefficients (r = 0.88**) between races 65 and 73, between races 65 and 3481(r = 0.64**), and between races 73 and 3481 (r = 0.73**). Resistance to the virulent Andean races 39 and 55 was detected on Pv02 (Fig 2). The disease pattern of both of these races was also strongly correlated (r = 0.82**). Resistance to both anthracnose races 7 and 109 resided on Pv04 (Fig 3). However, unlike the other races that were strongly correlated, no correlation between races 7 and 109 (r = -0.04; p = 0.55) was observed suggesting that either different loci on Pv04 confer resistance to these races or that resistance to race 7 within the ADP appears to reside on other chromosomes Pv10 and Pv11. The data suggest a correspondence with the major resistance genes *Co-1* on Pv01 [6] and with either the ANT02.1^UC^ QTL [21] or the *Co-u* gene on Pv02 [9], and putative new sources of resistance on Pv04, Pv10, and Pv11.

**Table 1:** Chromosome, position, p-value, proportion of phenotypic variation explained (R^2^) and minor allele frequency of the two most significant SNPs for seven races of anthracnose resistance measured on 226 Andean bean genotypes.

Anthracnose Race	SNP^a^	Chromosome	SNP Position (Mb)	P-value^b^	R^2^^c^	Minor Allele Frequency
7	ss715642306	Pv04	0.447165	6.37E-06	0.13	0.31
7	ss715649436	Pv04	0.487599	6.83E-06	0.12	0.31
39	ss715648452	Pv02	48.617342	4.60E-09	0.19	0.36
39	ss715648451	Pv02	48.606517	4.60E-09	0.19	0.36
55	ss715648451	Pv02	48.606517	3.26E-11	0.24	0.36
55	ss715648452	Pv02	48.617342	3.27E-11	0.24	0.36
65	ss715645251	Pv01	50.301532	4.04E-07	0.39	0.18
65	ss715646578	Pv01	48.340819	1.63E-06	0.14	0.37
73	ss715645251	Pv01	50.301532	3.44E-08	0.40	0.17
73	ss715645258	Pv01	50.155927	6.63E-07	0.16	0.28
109	ss715649432	Pv04	0.532194	3.88E-09	0.20	0.33
109	ss715640025	Pv04	0.212804	2.54E-05	0.12	0.33
3481	ss715645251	Pv01	50.301532	4.87E-10	0.47	0.19
3481	ss715646578	Pv01	48.340819	2.52E-05	0.10	0.36

^a^SNP = Single Nucleotide Polymorphic code

^b^P = significance level and E = exponential

^c^R^2^ = phenotypic variation explained by the SNP

GWAS did not prove informative for locating resistance within the ADP to race 2047 as only 10 lines were resistant to this virulent race (S3 Table). The failure to identify any putative resistance loci results from the low level of resistance (<5%) to race 2047 within the ADP and underscores the need to search for resistance outside the ADP. New sources of resistance *Co-12*, *Co-13*, *Co-14*, and *Co-15* genes [12, 44, 45, 46, 47] have recently been identified within the Andean gene pool. All the newly discovered resistance genes come from Brazilian landraces, and many condition resistance to anthracnose race 2047. The majority of anthracnose resistance genes in Andean beans reside on Pv01 and Pv04, making the identification of additional resistance sources on other chromosomes critical for future gene pyramiding [17, 18].

### Chromosome Pv01

The data on the location of resistance to races 65, 73, and 3481 indicate a correspondence with the *Co-1* gene on Pv01 (Fig 1), which is known to condition resistance to anthracnose races 65 and 73 [6]. Although no prior studies identified *Co-1* as conditioning resistance to race 3481, the *Co-1* gene is known to condition broad resistance [17] as race 3481 is not virulent on the differential cultivar Michigan Dark Red Kidney (MDRK). The *Co-1* gene is the known source of resistance in MDRK, as well as the popular resistance source A193 widely used in Mexican breeding programs [48]. The other differential cultivars of Andean origin that possess alleles at the *Co-1* locus are Kaboon with the *Co-1*^*2*^ allele, Perry Marrow with *Co-1*^*3*^ allele, AND277 with the *Co-1*^*4*^ allele, and Widusa with *Co-1*^*5*^ allele [17, 49, 50]. The *Co-1*^*4*^ allele which confers resistance to 21 different races of anthracnose including race 2047 did not appear to be prevalent in the ADP. Only 10 accessions within the panel showed resistance to race 2047 that is pathogenic on 11 of the 12 differential cultivars. Our data indicate that another locus or allele at the Co-1 locus must also be conditioning resistance to the virulent race 3481 within the ADP. It is likely that the locus conditioning resistance to race 3481 in the ADP is the same as the *Co-x* gene identified in Andean cultivar Jalo EEP558, which has been shown to condition resistance to the virulent *C*. *lindemuthianum* strain 100 [9]. Strain 100 corresponds with race 3993 when characterized using the binary numbering system, and is differentiated from race 3481 by its virulence on the differential cultivar TU (Binary no. 512 in differential series).

Resistance to races 65, 73, and 3481 were all associated significantly with SNP ss715645251, indicating that the region plays an important role in conferring anthracnose resistance. The SNP is located at 50.30 Mb on the physical map, which falls within the 58 kb region where the *Co-x* gene was shown to reside [24]. As such, the region around the SNP was investigated for potential positional gene candidates. The SNP fell within the exon of gene Phvul.001G243800, which codes for a leucine-rich repeat (LRR) receptor-like protein kinase that was also identified as one of eight candidate genes [24]. Kinases have previously been identified as playing an important role in the COK-4 anthracnose resistance locus on Pv08 [51, 52]. The identification of a LRR receptor-like protein kinases as a candidate gene, and its role in adaptive selection, supports prior literature indicating a co-evolution of common bean and anthracnose [53, 54].

The physical location of the *Co-1* gene identified in the GWAS on Pv01 in the ADP was validated in a Mesoamerican RIL population developed from a cross between two black bean cultivars, Jaguar and Puebla 152 differing in reaction to race 73. The two most significant SNPs identified in the ADP conferring resistance to race 73 were ss715645258 and ss715645251 at 50.16 and 50.30 Mb respectively. These two SNPs were also identified as highly significant within the RIL population, with the highest R^2^ values of all the significant SNPs (R^2^ = 69.4 and 67.8; S4 Table). Further, this region from 50.16 to 50.30 Mb overlaps with the region (50.10 to 50.22 Mb) identified with the highest LOD score (36.1) detected in the RIL population (S1 Fig). Since the Co-1 locus (original *A* gene) is widely reported in Andean bean germplasm [55] and has now been mapped in the ADP to the same region as the QTL for resistance in Jaguar, we conclude that the gene conditioning resistance to race 73 in the Mesoamerican black bean cultivar Jaguar is the same *Co-1* gene as was detected in the ADP. In addition, the region identified as the location of the *Co-x* gene in Jalo EEP558 falls within the 50.2 to 50.3 Mb region identified as the location of the *Co-1*^*2*^ allele in the navy bean cultivar ‘Bolt’ [25]. Additional evidence that the *Co-x* and *Co-1* are the same gene comes from genetic studies where no segregation was detected in an allelism test of 200 progeny from the F_2_ population of MDRK (*Co-1*) x Jalo EEP558 (*Co-x*) inoculated with race 73 [56].

Given the importance of this region we choose to develop an InDel marker linked to the *Co-1* gene for use in breeding programs that do not have access to SNP technology. A total of 50 InDel markers were screened against the bulks and parents, but only 12 were polymorphic. The closest InDel to the *Co-1* gene was NDSU_IND_1_50.2219 and this marker was approximately 600 bp from the ss715645252 SNP at 50.22 Mb. The InDel co-segregated with the resistant and susceptible reactions of 95 RILs except in three RILs where recombination between marker and phenotype was observed (64R+:28S-:1R-:2S+). The InDel produced amplicon of ~150 bp in Puebla 152 (susceptible) parent and ~170 bp in the resistant parent Jaguar (S2 Fig) and mapped at 3.1cM from the gene. The InDel marker (NDSU_IND_1_50.2219) was validated by screening 30 cultivars and advanced breeding lines from the MSU breeding program, some known to possess alleles at the Co-1 locus. The 30 genotypes were screened with race 73 and grouped into three categories of resistant, susceptible or segregating (Table 2). The InDel marker co-segregated with the phenotypic data and identified all resistant alleles except *Co-1*^*5*^ at the Co-l locus including the *Co-x* in Jalo EEP558. Two heterogeneous genotypes N12447 and B11363 that segregated against race 73 produced two bands of 150 and 170 bp confirming their heterozygosity at the *Co-1* locus (S2 Fig) and the broad utility of the InDel marker for marker assisted selection for individual alleles at the Co-1 locus.

**Table 2:** Phenotypic and genotypic reaction to screening RIL parents, differentials, cultivars and advanced breeding lines to anthracnose race 73 and InDel Marker NDSU_IND_1_50.2219.

Gel Number^a^	Genotype	Gene Pool^b^	Anthracnose allele^c^	Phenotypic reaction^d^	Genotypic reaction^e^
1	Zenith	MA	*Co-1/Co-1*	R	AA
2	B12724	MA	*Co-1/Co-1*	R	AA
3	B14302	MA	*Co-1/Co-1*	R	AA
4	B14303	MA	*Co-1/Co-1*	R	AA
5	B14311	MA	*co-1/co-1*	S	BB
6	K14104	A	*Co-1/Co-1*	R	AA
7	K12803	A	*Co-1/Co-1*	R	AA
8	K14807	A	*Co-1/Co-1*	R	AA
9	N14230	MA	*Co-1/Co-1*	R	AA
10	N13131	MA	*Co-1/Co-1*	R	AA
11	N13140	MA	*Co-1/co-1*	H	AA
12	N12447	MA	*Co-1/co-1*	H	AB
13	B11363	MA	*Co-1/co-1*	H	AB
14	Alpena	MA	*co-1/co-1*	S	BB
15	Zorro	MA	*co-1/co-1*	S	BB
16	Snowdon	A	*Co-1/Co-1*	R	AA
17	K11306	A	*Co-1/Co-1*	R	AA
18	K11714	A	*Co-1/Co-1*	R	AA
19	K11707	A	*Co-1/Co-1*	R	AA
20	K11320	A	*Co-1/Co-1*	R	AA
21	Rosetta	MA	*co-1/co-1*	S	BB
22	Puebla 152	MA	*co-1/co-1*	S	BB
23	Jaguar	MA	*Co-1/Co-1*	R	AA
24	Jalo EEP558	A	*Co-1*, *Co-x*	R	AA
25	BAT 93	MA	*co-1/co-1*	S	BB
26	AND277	A	*Co-1*^*4*^*/ Co-1*^*4*^	R	AA
27	MDRK	A	*Co-1/Co-1*	R	AA
28	Kaboon	A	*Co-1*^*2*^*/ Co-1*^*2*^	R	AA
29	Perry Marrow	A	*Co-1*^*3*^*/ Co-1*^*3*^	R	AA
30	Widusa	A	*Co-1*^*5*^*/ Co-1*^*5*^	R	BB

^a^gel number shown in S2 Fig

^b^ MA = Mesoamerica, A = Andean gene pool of host genotypes]

^c^anthracnose resistance alleles at the Co-1locus

^d^disease reaction to race 73 based on six plants: R = Resistant; S-Susceptible, H = Heterozygous

^e^genotypes screened with NDSU_IND-1_50.2219 linked to *Co-1* anthracnose gene; AA = Homozygous resistant; BB = Homozygous susceptible; AB = Heterozygous.

### Chromosome Pv02

The data from GWAS indicate that resistance to anthracnose races 39 and 55 resides on Pv02 (Fig 2) and appears to be associated with either the QRL ANT02.1^UC^ [21] or the *Co-u* gene on Pv02 [9]. A major QRL ANT02.1^UC^ was identified as the source conferring resistance to races 38 and 55 in the Brazilian carioca bean genotype ICA-UNA [21]. In that study the molecular marker IAC255 was tightly linked to the QRL, which was less than 1 Mb away from SNP ss715648451, located at 48.61 Mb on the physical map. SNP ss715648451was the most significant SNP identified for resistance to both races 39 and 55 in the current study. This is a significant finding as both races 39 and 55 are aggressive Andean races, and as such, identifying new sources of resistance within Andean beans is critical for future breeding efforts. Previously, the *Co-u* gene conditioning resistance to Tanzanian strains E4 and E42b in genotype BAT93 was reported in the vicinity of the *I* gene on Pv02 [9,11]. In the absence of a physical map position for the *Co-u* gene we were not able to verify a direct association with resistance locus reported here. However, the most tightly linked SNPs (ss715648451, ss715648452, ss71639906) associated with resistance to races 39 and 55 in our study were recently mapped [57] at 50 kb from the cluster of NBS-LRR R-genes residing near the *I* gene. Data would suggest that resistance identified to races 39 and 55 in the ADP is in fact the *Co-u* gene in the Mesoamerica genotype BAT93 previously reported [9]. The most significant SNP ss715648451 was located within the exon of gene Phvul.002G328300, which codes for a mitogen-activated protein kinase, which are known to play a role in disease response in *Arabidopsis* [58]. Therefore, this gene could play an important role in initiating disease response in common bean. A second locus on Pv02 was recently reported to condition resistance to races 3, 19 and 449 in the Andean cultivar Xana [10] but based on a map position of 40.37–42.52 Mb, the two loci appear to be separate regions of Pv02.

### Chromosome Pv04

The data from GWAS indicated that resistance to anthracnose races 7 and 109 in the ADP resides within an 85 kb region on Pv04. The most significant SNP for race 7 was ss715642306 at 0.45 Mb, while the most significant SNP associated with race 109 was ss715649432 located at 0.53 Mb. Further, there was no correlation between resistance to anthracnose races 7 and 109 (r = -0.04), indicating that the two sources of resistance could be separate resistance genes. Another factor that prevented a correlation between the resistance of race 7 and 109 is that race 7 also showed additional levels of resistance on Pv10 and Pv11. One or both sources of resistance could be associated with the major Co-3 locus on Pv04 (Fig 3). The role of Co-3 locus in anthracnose resistance is well documented in the literature [6], and five alleles have been identified in members of the differential series [11]. These include Mexico 222 (*Co-3*), Mexico 227 now extant (*Co-3*^*2*^), PI 207262 (*Co-3*^*3*^), BAT93 (*Co-3*^*3*^), Ouro Negro (*Co-3*^*4*^) and MSU-7 (*Co-3*^*5*^) [19]. The *Co-3*^*4*^ allele was previously mapped near the genomic marker g2303 located at 3.36 Mb on the physical map [47], which is distant from the region identified in this study. Further, *Co-3*^*4*^ was identified as conferring resistance to race 7. Additionally, the Andean *Co-z* resistance gene has been identified distally to the *Co-3*^*4*^ [59], and could potentially play a role in resistance within the ADP. The Andean *Co-15* gene in the Brazilian landrace Corinthiano is tightly linked to the genomic marker g2685 located at 9.08 Mb on Pv04 [12]. However, the resistance identified in the ADP is not likely to be the *Co-15* gene as *Co-15* has been shown to confer resistance to race 2047, which was highly virulent on 215/226 entries included in the study (S3 Table). The results of this study are important for developing future breeding strategies in Andean beans as previous work on *Co-3* gene and its alleles has been largely reported in Mesoamerican germplasm.

Unlike resistance loci previously mapped on Pv01 and Pv02, the significant SNPs associated with resistance to races 7 and 109 were not the same. In the case of race 7, SNPs ss715642306 and ss715649436 were identified as significant. The first SNP falls within the gene Phvul.004G005800 while the later SNP is 2 kb from the gene Phvul.004G006300. Both genes encode for Cytochrome P450. Cytochromes are known for playing a role in enzymatic complexes that catalyze redox reactions [60], which can trigger the plant hypersensitive disease resistance response [61]. Alternatively, SNP ss715649432 was identified as most strongly associated with resistance to race 109, which was also identified as a significant SNP for resistance to race 73. The SNP fell within the gene Phvul.004G006800, which encodes for the glycoprotein gp210 component of the nuclear pore complex. Although not as well recognized as other components of disease response, the nuclear pore complex has been found to condition disease resistance in *Arabidopsis* [62].

### Chromosome Pv10

The data from GWAS indicates moderate levels of resistance to anthracnose race 7 on Pv10, although not substantial enough to be considered significant with the conservative Bonferonni corrected p-value (Fig 3). No major Co genes have been previously identified on Pv10, other than a QTL for resistance to race 7 but the location was not fine mapped [63]. The region near the most strongly linked SNP ss715648754 (p = 1.77 x 10^−4^), located at 3.78 Mb, was investigated for potential positional gene candidates. The SNP fell 1.2 kb from the gene Phvul.010G025500, which encodes for an N-terminal Toll/interleukin-1 receptor (TIR)-like domain (TNLs). This gene is one of many genes identified near the end of Pv10 in a dense cluster of resistance-associated genes in the recently sequenced Andean genotype G19833 [23].

### Chromosome Pv11

Results indicate that resistance to race 7 in the ADP is also present on Pv11, although not significant enough to be recognized with the conservative Bonferonni corrected p-value (Fig 3). Prior support exists for a QTL on Pv11 for resistance to race 385 (Cl43) in the Andean accession G19833 [63]. This region appears to be distinct from the *Co-2* gene cluster that is a common source of resistance to race 7 in Mesoamerican beans [6]. Using BLASTN on Phytozome v10.2, the genetic marker SCAreoli [64] linked to the *Co-2* gene, was located at 39.73 Mb whereas the ss715645476 SNP identified in the ADP on Pv11 was located at 1.69 Mb. The most strongly linked SNP ss715645476 (p = 2.14 x 10^−5^), was located within the gene Phvul.011G021500, which encodes for a Phospholipid scramblase. The activity of this enzyme has been suggested to result in the outward translocation of phosphatidylserine from the cell, a major signal for macrophages to eliminate apoptotic cells that is known to interact with cytochrome C [65]. It seems probable that this gene could play a complementary role in resistance to race 7 as the gene candidate on Pv04 encodes for cytochrome P450 suggesting that both genes are involved in programmed cell death to prevent further spread of disease. Support comes from prior genetic studies which show complementary gene action for resistance to race 7 in the common bean cultivars Xana and Cornell 49242 on Pv04 and Pv11 [10].

## Conclusions

Information on resistance in this diverse group of Andean bean lines will be useful in future breeding efforts to develop anthracnose resistant cultivars depending on the prevailing races in a region. Finding resistance in adapted Andean lines with favorable agronomic and seed traits could have important implications and applications for breeders within target countries. Not only does it help maintain bean diversity through additional resistance options, but it also allows for a more rapid introgression of resistance into future Andean bean cultivars. A lack of information on the physical position of markers linked to the major resistance genes in the published literature prevents a final determination of co-localization between results from the GWAS and the presumed location of many anthracnose resistance genes. In this study, new sources of anthracnose resistance in Andean beans were discovered on Pv02, Pv10, and Pv11, as well as a unique location on Pv04. Breeders will need to identify the most effective resistance gene or allele at these loci prior to pyramiding genes from different chromosomes for more durable resistance. The physical position and the candidate genes identified in the current study will serve as a basis for developing functional markers to facilitate this effort. The resistance deployed in the MSU breeding program has largely been assumed to be controlled by the *Co-1* gene and that assumption was confirmed in this study. A major putative QTL for resistance to anthracnose in both Andean and Mesoamerican beans was identified on Pv01 adjacent to SNPs ss715645251 at 50.30 Mb within the 58 kb region (50.26–50.32 Mb) where the *Co-x* was mapped. It is likely that this region corresponds to the major *Co-1* resistance cluster, including the *Co-x* resistance gene. The identification of an InDel marker (50.22 Mb) tightly linked to four alleles at the Co-1 locus will be especially useful for the continued effort of breeders in developing countries as it can be utilized for marker assisted breeding in labs where resources are limiting.

## Supporting Information

S1 Fig Bean chromosome Pv01 in which anthracnose race 73 resistance was directly located with graphical representation of LOD values in black bean RIL population; AR = anthracnose resistance. (TIF)

S2 Fig Validation of NDSU_IND_1_50.2219 InDel marker for the presence of anthracnose resistance gene Co-1 in commercial bean cultivars, differential genotypes and advanced breeding lines. M = 100bp ladder and lanes 1 to 30 are common bean genotypes: 1 = Zenith; 2 = B12724; 3 = B14302; 4 = B14303; 5 = B14311; 6 = K14104; 7 = K12803; 8 = K14807; 9 = N14230; 10 = N13131; 11 = N13140; 12 = N12447; 13 = B11363; 14 = Alpena; 15 = Zorro; 16 = Snowdon; 17 = K11306; 18 = K11714; 19 = K11707; 20 = K11320; 21 = Rosetta; 22 = Puebla 152; 23 = Jaguar; 24 = JaloEEP558; 25 = BAT93; 26 = AND277; 27 = MDRK; 28 = Kaboon; 29 = Perry Marrow; 30 = Widusa.
(TIF)

S1 Table Results from screening of 226 Andean lines from the Andean Diversity Panel against eight races of *Colletotrichum lindemuthianum*. ^a^Disease scores are mean of 6 plants, 0 are most resistant, 5 are most susceptible. Cells in which an ‘h’ is listed is indication of a heterogeneous mixture. Further details on the origin of the lines in the ADP can be found in the literature [27].
(DOCX)

S2 Table Twenty-eight lines within the 226 accessions from the Andean Diversity Panel with resistance to six or more of the eight races of anthracnose included in the study. ^a^Disease scores are mean of six plants, 0 are most resistant, 5 are most susceptible [30].
(DOCX)

S3 Table The gene pool and prevalence of anthracnose resistance to eight races of *Colletotrichum lindemuthianum* within the 226 bean lines from the Andean Diversity Panel. ^a^A, Andean, MA, Mesoamerican or Mx Mixed gene pool designation of the race is based on level of virulence on host differentials from either gene pool; Further information on races is provided in the literature [6, 28].
(DOCX)

S4 Table Thirteen SNP markers, location and physical position on chromosome Pv01 used to identify major QTL for resistance to anthracnose race 73 in Jaguar x Puebla 152 black bean RIL population. **significant at Pr(F) = 0.01%; further information on SNP can be found in literature [32].
(DOCX)

## References

[1] Broughton WJ, Hernández G, Blair M, Beebe S, Gepts P, Vanderleyden J. Beans (*Phaseolus* spp.)–model food legumes. Plant Soil. 2003; 252: 55–128.

[2] Gepts PF, Aragão JL, de Barros E, Blair MW, Brondani R, Broughton W, et al Genomics of Phaseolus Beans, a Major Source of Dietary Protein and Micronutrients in the Tropics In: Moore P.H. and Ming R. (Eds.), Genomics of Tropical Crop Plants. 2008; pp. 113–143. Springer Publishing, New York, NY.

[3] Graham PH, Vance CP. Legumes: importance and constraints to greater use. Plant Physiol. 2003; 131: 872–877 12644639 10.1104/pp.017004 PMC1540286

[4] Melotto M, Balardin RS, Kelly JD. Host-pathogen interaction and variability of *Colletotrichum lindemuthianum* In: Prusky D., Freeman S., and Dickman M.B. (Eds.), *Colletotrichum* Host Specificity, Pathology, Host–Pathogen Interaction. 2000; pp. 346–361. APS Press, St. Paul, MN.

[5] Guzman P, Donado MR, Galvez GE. Perdidas economicas causadas por la antracnosis del frijol *Phaseolus vulgaris* en Colombia. Turrialba. 1979; 29: 65–67

[6] Ferreira JJ, Campa A, Kelly JD. Organization of genes conferring resistance to anthracnose in common bean In: Varshney R.K. and Tuberosa R. (eds.) Translational Genomics for Crop Breeding, Volume I: Biotic Stresses. 2013; pp. 151–181. John Wiley & Sons, Inc, Ames, IA

[7] Kelly JD, Vallejo VA. A comprehensive review of the major genes conditioning resistance to anthracnose in common bean. Hortscience. 2004; 39: 1196–1207

[8] Pastor-Corrales MA, Tu JC. Anthracnose p. 77–104. In Schwartz H.F. and Pastor-Corrales M.A. (ed) Bean production problems in the tropics. 1989; pp. 77–104. Centro Internacional de Agricultura Tropical (CIAT), Cali, Colombia

[9] Geffroy V, Sévignac M, Billant P, Dron M, Langin T. Resistance to *Colletotrichum lindemuthianum* in *Phaseolus vulgaris*: a case study for mapping two independent genes. Theor Appl Genet. 2008; 116: 407–415 18060540 10.1007/s00122-007-0678-y

[10] Campa A, Rodriguez-Suárez C, Giraldez R, Ferreira JJ. Genetic analysis of the response to eleven *Colletotrichum lindemuthianum* races in a RIL population of common bean (*Phaseolus vulgaris* L.). BMC Plant Biology. 2014; 14: 115 10.1186/1471-2229-14-115 24779442 PMC4021056

[11] Meziadi C, Richard MMS, Derquennes A, Thareau V, Blanchet S, Gratias A, et al Development of molecular markers linked to disease resistance genes in common bean based on whole genome sequence. Plant Sci. 2016; 242: 351–357 10.1016/j.plantsci.2015.09.006 26566851

[12] Sousa LL, Goncalves AO, Gonçalves-Vidigal MC, Lacanallo GF, Fernandez AC, Awale HE, et al Genetic characterization and mapping of anthracnose resistance of Corinthiano common bean landrace cultivar. Crop Sci. 2015; 55: 1–11

[13] Coelho RT, Gonçalves-Vidigal MC, Vidigal Filho PS, Lacanallo GF, Darben LM, Silva CR, et al Characterization of the anthracnose resistance gene in the Mesoamerican common bean cultivar Crioulo 159. Ann Rep Bean Improv Coop. 2013; 56: 43–44

[14] Coimbra GK, Gonçalves-Vidigal MC, Tessaro R, Sousa LL, Lacanallo GF, Silva V, et al. Mapping of the Co-16 resistance gene to *Colletotrichum lindemuthianum* in the Crioulo 159 cultivar. Presented at 2014 Conafe Congress, Brazil http://www.conafe2014.com.br/_apps/trabalhos/326/326_1.doc

[15] Lacanallo GF, Gonçalves-Vidigal MC. Mapping of an Andean gene for anthracnose resistance (Co-13) in common bean (*Phaseolus vulgari*s L.) Jalo Listras Pretas landrace. Aust J Crop Sci. 2015; 9:394–400

[16] Trabanco N, Campa A, Ferreira JJ. Identification of a new chromosomal region involved in the genetic control of resistance to anthracnose in common bean. The Plant Genome. 2015; 8: 10.3835/plantgenome2014.10.0079 33228300

[17] Gonçalves-Vidigal MC, Cruz AS, Garcia A, Kami J, Vidigal Filho PS, Sousa LL, et al Linkage mapping of the Phg-1 and Co-1^4^ genes for resistance to angular leaf spot and anthracnose in the common bean cultivar AND 277. Theor Appl Genet. 2011; 122: 893–903 10.1007/s00122-010-1496-1 21113774 PMC3043234

[18] Gonçalves-Vidigal MC, Cruz AS, Lacanallo GF, Vidigal Filho PS, Sousa LL, Pacheco CMNA, et al Co-segregation analysis and mapping of the anthracnose Co-10 and angular leaf spot Phg-ON disease-resistance genes in the common bean cultivar Ouro Negro. Theor Appl Genet. 2013; 126:2245–2255 10.1007/s00122-013-2131-8 23760652

[19] Sousa LL, Cruz AS, Vidigal Filho PS, Vallejo VA, Kelly JD, Gonçalves-Vidigal MC. Genetic mapping of the resistance allele Co-5^2^ to *Colletotrichum lindemuthianum* in the common bean MSU 7–1 line. Aust J Crop Sci. 2014; 8:317–323

[20] Valentini G, Gonçalves-Vidigal MC, Cregan PB, Song Q, Pastor-Corrales MA. Using SNP genetic markers to elucidate the linkage of the Co-3^4^/Phg-3 anthracnose and angular leaf spot resistance gene cluster with the Ur-14 rust resistance gene. Ann Rep Bean Improv Coop. 2015; 58:21–22

[21] Oblessuc PR, Baroni RM, da Silva Pereira G, Chioratto AF, Carbonell SAM, Briñez B, et al Quantitative analysis of race-specific resistance to *Colletotrichum lindemuthianum* in common bean. Mol Breed. 2014; 34:1313–1329. 10.1007/s11032-014-0118-z

[22] González AM, Yuste-Lisbona FJ, Rodiño AP, De Ron AM, Capel C, García-Alcázar M, et al Uncovering the genetic architecture of *Colletotrichum lindemuthianum* resistance through QTL mapping and epistatic interaction analysis in common bean. Front Plant Sci. 2015; 6: 141 10.3389/fpls.2015.00141 25852706 PMC4362272

[23] Schmutz J, McClean PE, Mamidi S, Wu GA, Cannon SB, Grimwood J, et al A reference genome for common bean and genome-wide analysis of dual domestications. Nature Genetics. 2014; 46: 707–713 10.1038/ng.3008 24908249 PMC7048698

[24] Richard MMS, Pflieger S, Sévignac M, Thareau V, Blanchet S, Li Y, et al Fine mapping of Co-x, an anthracnose resistance gene to a highly virulent strain of *Colletotrichum lindemuthianum* in common bean. Theor Appl Genet. 2014; 127: 1653–1666 10.1007/s00122-014-2328-5 24859268

[25] Vazin M, Burt AJ, Zarei A, Xie W, Pauls KP, Gillard C, et al Molecular characterization of anthracnose resistance to race 73 in the navy bean variety Bolt. Ann Rep Bean Improv Coop. 2014; 57: 161–162

[26] Oblessuc PR, Francisco C, Melotto M. The Co‑4 locus on chromosome Pv08 contains a unique cluster of 18 COK‑4 genes and is regulated by immune response in common bean. Theor Appl Genet. 2015; 128:1193–1208. 10.1007/s00122-015-2500-6 25805316

[27] Cichy KA, Porch T, Beaver JS, Cregan PB, Fourie D, Glahn R, et al A *Phaseolus vulgaris* diversity panel for Andean bean improvement. Crop Sci. 2015; 55:2149–2160. 10.2135/cropsci2014.09.0653

[28] Balardin RS, Jarosz AM, Kelly JD. Virulence and molecular diversity in *Colletotrichum lindemuthianum* from South, Central, and North America. Phytopathology. 1997; 87: 1184–1191 10.1094/PHYTO.1997.87.12.1184 18945016

[29] Kelly JD, Afanador L, Cameron L. New races of *Colletotrichum lindemuthianum* in Michigan and implications in dry bean resistance breeding. Plant Dis. 1994; 78: 892–894

[30] Drijfhout E, Davis JHC. Selection of a new set of homogeneously reacting bean (*Phaseolus vulgaris*) differentials to differentiate races of *Colletotrichum lindemuthianum*. Plant Path. 1989; 38: 391–396

[31] Doyle JJ. A rapid DNA isolation procedure for small quantities of fresh leaf tissue. Phytochemical Bull. 1987; 19: 11–15

[32] Song Q, Jia G, Hyten DL, Jenkins J, Hwang E-Y, Schroeder S, et al SNP assay development for linkage map construction, anchoring whole genome sequence and other genetic and genomic applications in common bean. G3: Genes| Genomes| Genetics. 2015; 5:2285–2290, 10.1534/g3.115.020594 26318155 PMC4632048

[33] Price A, Patterson N, Plenge R, Weinblatt M, Shadick N, Reich D. Principle components analysis corrects for stratification in genome-wide association studies. Nature Genetics. 2006; 38: 904–909 16862161 10.1038/ng1847

[34] Kamfwa K, Cichy KA, Kelly JD. Genome-wide association study of agronomic traits in common bean. The Plant Genome. 2015; 8: 10.3835/plantgenome2014.09.0059 33228312

[35] Zhang Z, Ersoz E, Lai C-Q, Todhunter RJ, Tiwari HK, Gore MA, et al Mixed linear model approach adapted for genome-wide association studies. Nature Genetics. 2010; 42: 355–360 10.1038/ng.546 20208535 PMC2931336

[36] Goodstein DM, Shu S, Howson R, Neupane R, Hayes RD, Fazo J, et al Phytozome: a comparative platform for green plant genomics. Nucleic Acids Res. 2012; 40: 1178–1186 10.1093/nar/gkr944 PMC3245001 22110026

[37] Kelly JD, Hosfield GL, Varner GV, Uebersax MA, Taylor J. Registration of ‘Jaguar’ black bean. Crop Sci. 2001; 51: 1647–1648

[38] Van Ooijen JW. JoinMap 4: Software for the calculation of genetic linkage maps in experimental populations 2006; Kyazma BV , Wageningen, Netherlands

[39] Wang S, Basten CJ, Zeng Z-B. Windows QTL cartographer 2.5. 2012; Department of Statistics, North Carolina State University, Raleigh, North Carolina

[40] Voorrips RE. MapChart: software for the graphical presentation of linkage maps and QTLs. J Hered. 2002; 93: 77–78 12011185 10.1093/jhered/93.1.77

[41] Moghaddam SM, Song Q, Mamidi S, Schmutz J, Lee R, Cregan P, et al Developing market class specific InDel markers from next generation sequence data in *Phaseolus vulgaris* L. Front Plant Sci. 2014; 5:185 10.3389/fpls.2014.00185 PMC4026720 24860578

[42] Kelly JD, Hosfield GL, Varner GV, Uebersax MA, Long RA, Taylor J. Registration of 'Red Hawk' dark red kidney bean. Crop Sci. 1998; 38:280–281

[43] Fivawo NC, Msolla SN. The diversity of common bean landraces in Tanzania. Tanzania J Nat Appl Sci. 2011; 2: 337–351

[44] Gonçalves AMO, Gonçalves-Vidigal MC, Vidigal Filho PS, Polentine JP, Lacanallo GF, Coimbra GK. Characterization of the anthracnose resistance gene in Andean common bean Corinthiano cultivar. Ann Rep Bean Improv Coop. 2010; 53: 220–221

[45] Gonçalves-Vidigal MC, Lacanallo GF, Vidigal Filho PS. A new Andean gene conferring resistance to anthracnose in common bean (*Phaseolus vulgaris* L.) cultivar Jalo Vermelho. Plant Breed. 2008; 127: 592–596

[46] Gonçalves-Vidigal MC, Vidigal Filho PS, Medeiros AF, Pastor-Corrales MA. Common bean landrace Jalo Listras Prestas is the source of a new Andean anthracnose resistance gene. Crop Sci. 2009; 49: 133–138

[47] Gonçalves-Vidigal MC, Meirelles AC, Poletine JP, Sousa LL, Cruz AS, Nunes MP, et al Genetic analysis of anthracnose resistance in Pitanga dry bean cultivar. Plant Breed. 2012; 131: 423–429

[48] Mendoza A, Hernández F, Hernández S, Ruíz D, de la Vega OM, Mora G, et al Identification of Co-1 anthracnose resistance and linked molecular markers in common bean line A193. Plant Dis. 2001; 85: 252–255 10.1094/PDIS.2001.85.3.252 30832037

[49] Gonçalves-Vidigal MC, Kelly JD. Inheritance of anthracnose resistance in the common bean cultivar Widusa. Euphytica. 2006; 151: 411–419

[50] Melotto M, Kelly JD. An allelic series at the Co-1 locus conditioning resistance to anthracnose in common bean of Andean origin. Euphytica. 2000; 116: 143–149

[51] Melotto M, Coelho MF, Pedrosa-Harand A, Kelly JD Camargo LEA. The anthracnose resistance locus Co-4 of common bean is located on chromosome 3 and contains putative disease resistance-related genes. Theor Appl Genet. 2004; 109: 690–699 15221144 10.1007/s00122-004-1697-6

[52] Burt AJ, William HM, Perry G, Khanal R, Pauls KP, Kelly JD, et al Candidate gene identification with SNP marker-based fine mapping of anthracnose resistance gene Co-4 in common bean. PLoS ONE. 2015;10(10): e0139450 10.1371/journal.pone.0139450 26431031 PMC4592015

[53] Balardin RS, Kelly JD. Interaction between *Colletotrichum lindemuthianum* races and gene pool diversity in *Phaseolus vulgaris*. HortScience. 1998; 123: 1038–1047

[54] Geffroy V, Sicard D, de Oliveira JCF, Sévignac M, Cohen S, Gepts P, et al Identification of an ancestral resistance gene cluster involved in the coevolution process between *Phaseolus vulgaris* and its fungal pathogen *Colletotrichum lindemuthianum*. Mol Plant-Microbe Int. 1999; 12: 774–784 10.1094/MPMI.1999.12.9.774 10494630

[55] Young RA, Kelly JD. Is the anthracnose resistance 'A' gene the same in cultivars belonging to both bean gene pools. Ann Rep Bean Improv Coop. 1996; 39:296–297

[56] Vallejo VA, Awale HE, Kelly JD. Characterization of the anthracnose resistance in the Andean bean cultivar Jalo EEP558. Ann Rep Bean Improv Coop. 2003; 44: 121–122

[57] Bello MH, Moghaddam SM, Massoudi M, McClean PE, Cregan PB, Miklas PN. Application of in silico bulked segregant analysis for rapid development of markers linked to bean common mosaic virus resistance in common bean. BMC Genomics. 2014; 15: 903 10.1186/1471-2164-15-903 25326146 PMC4210609

[58] Menke FLH, van Pelt JA, Pieterse CMJ, Klessig DF. Silencing of the mitogen-activated protein kinase MPK6 comprises disease resistance in *Arabidopsi*s. The Plant Cell. 2004; 16: 897–907 15020743 10.1105/tpc.015552 PMC412864

[59] Geffroy V, Sévignac M, De Oliveira JCF, Fouilloux G, Skroch P, Thoquet P, et al Inheritance of partial resistance against *Colletotrichum lindemuthianum* in *Phaseolus vulgaris* and co-localization of quantitative trait loci with genes involved in specific resistance. Mol Plant-Microbe Int. 2000; 13: 287–296 10.1094/MPMI.2000.13.3.287 10707354

[60] Grant JJ, Loake GJ. Role of reactive oxygen intermediates and cognate redox signaling in disease resistance. Plant Physiol. 2000; 124: 21–29 10982418 10.1104/pp.124.1.21 PMC1539275

[61] Delledonne M, Zeier J, Marocco A, Lamb C. Signal interactions between nitric oxide and reactive oxygen intermediates in the plant hypersensitive disease resistance response. Proc Nat Acad Sci. 2001; 98: 13454–13459 11606758 10.1073/pnas.231178298 PMC60892

[62] Cheng YT, Germain H, Wiermer M, Bi D, Xu F, García AV, et al Nuclear pore complex component MOS7/Nup88 is required for innate immunity and nuclear accumulation of defense regulators in Arabidopsis. The Plant Cell. 2009; 21: 2503–2516 10.1105/tpc.108.064519 19700630 PMC2751965

[63] López CE, Acosta IF, Jara C, Pedraza F, Gaitán-Solís E, Gallego G, et al Identifying resistance gene analogs associated with resistances to different pathogens in common bean. Phytopathology. 2003; 93: 88–95 10.1094/PHYTO.2003.93.1.88 18944161

[64] Creusot F, Macadré C, Ferrier Cana E, Riou C, Geffroy V, Sévignac M, et al Cloning and molecular characterization of three members of the NBS-LRR subfamily located in the vicinity of the Co-2 locus for anthracnose resistance in *Phaseolus vulgaris*. Genome. 1999; 42: 254–264 10231959 10.1139/g98-134

[65] Collazo C, Chacón O, Borrás O. Programmed cell death in plants resembles apoptosis of animals. Biotecnología Aplicada. 2006; 23: 1–10

