# Supplementary figures and images for: Genome-Wide Association Study of Anthracnose Resistance in Andean Beans (Phaseolus vulgaris)
Source: PLoS One. 2016 Jun 6;11(6):e0156391. doi: 10.1371/journal.pone.0156391 (PMC4894742; doi:10.1371/journal.pone.0156391)

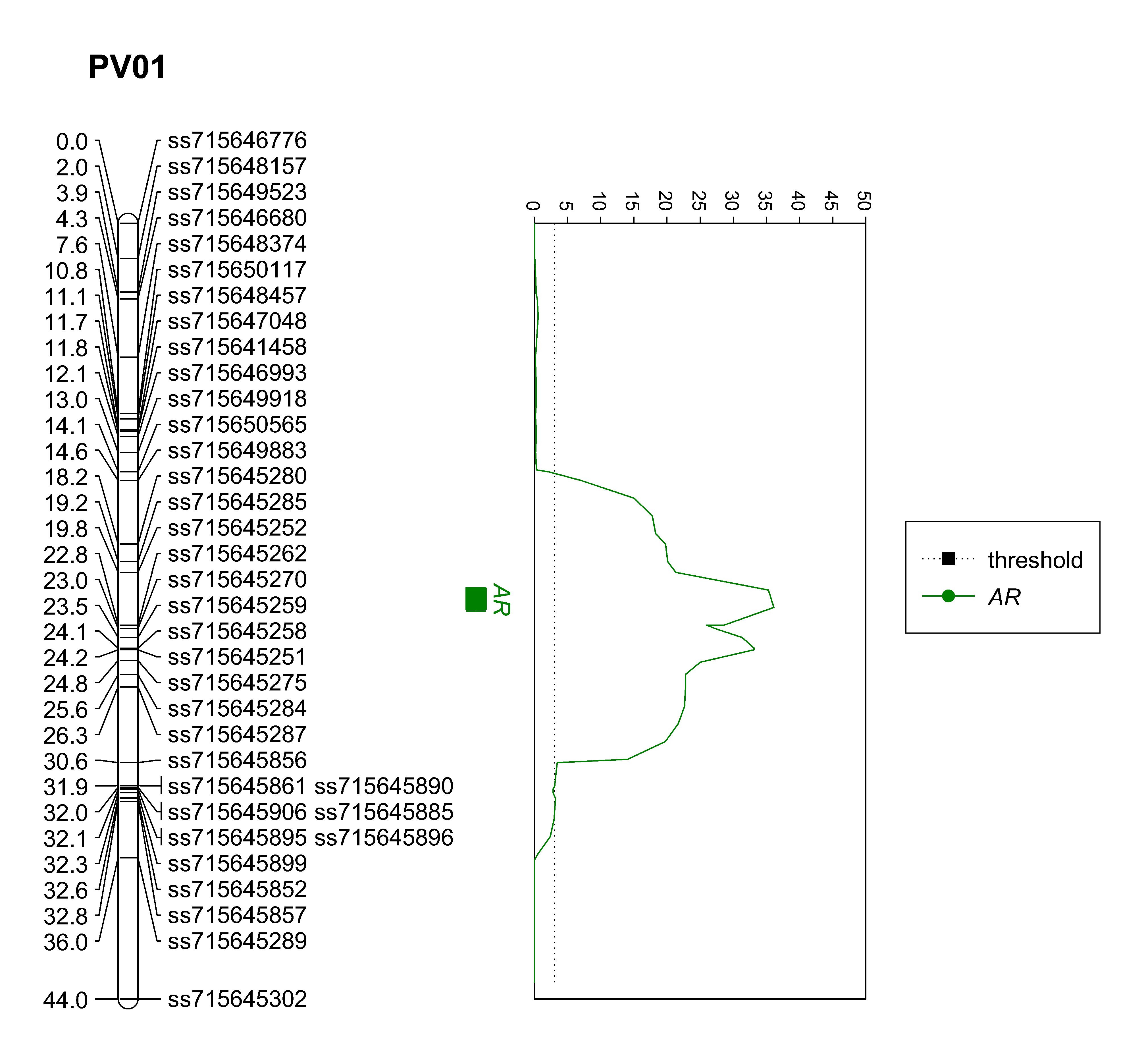

Supplement: S1 Fig — (TIF) [file pone.0156391.s001.tif]

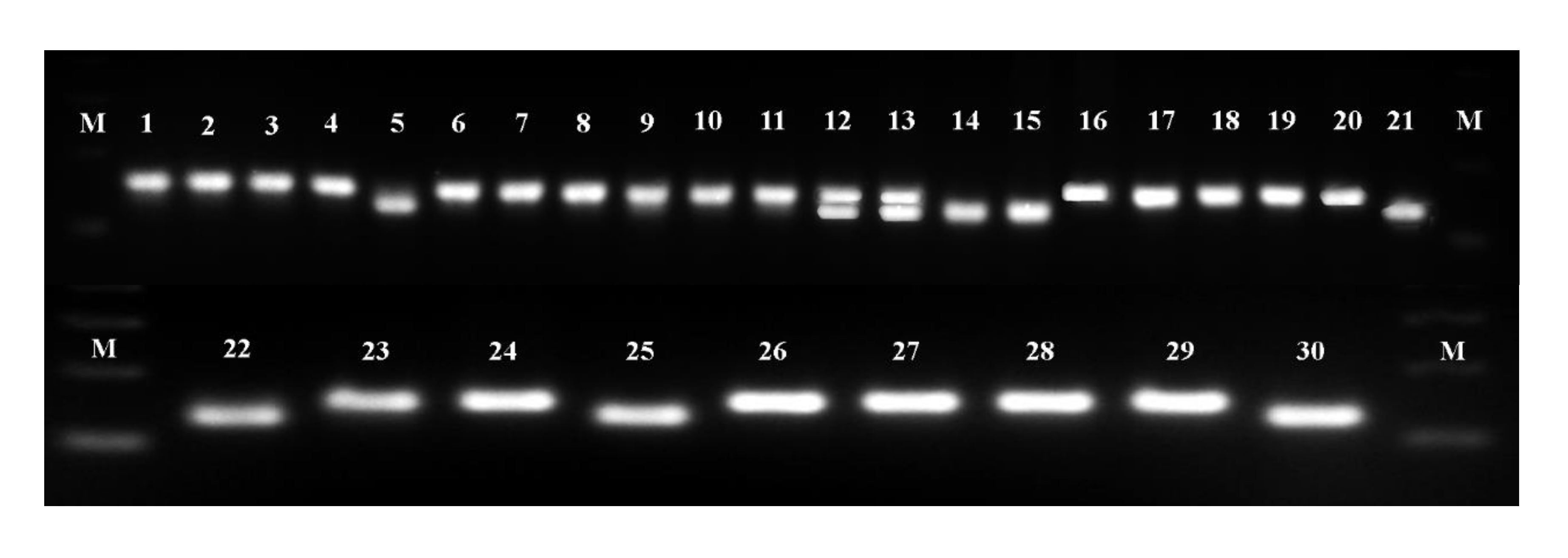

Supplement: S2 Fig — M = 100bp ladder and lanes 1 to 30 are common bean genotypes: 1 = Zenith; 2 = B12724; 3 = B14302; 4 = B14303; 5 = B14311; 6 = K14104; 7 = K12803; 8 = K14807; 9 = N14230; 10 = N13131; 11 = N13140; 12 = N12447; 13 = B11363; 14 = Alpena; 15 = Zorro; 16 = Snowdon; 17 = K11306; 18 = K11714; 19 = K11707; 20 = K11320; 21 = Rosetta; 22 = Puebla 152; 23 = Jaguar; 24 = JaloEEP558; 25 = BAT93; 26 = AND277; 27 = MDRK; 28 = Kaboon; 29 = Perry Marrow; 30 = Widusa. (TIF) [file pone.0156391.s002.tif]
