# Supplementary material for: Genome-Wide Association Study of Anthracnose Resistance in Andean Beans (Phaseolus vulgaris)
Source: PLoS One. 2016 Jun 6;11(6):e0156391. doi: 10.1371/journal.pone.0156391 (PMC4894742; doi:10.1371/journal.pone.0156391)
Supplement: S1 Table — aDisease scores are mean of 6 plants, 0 are most resistant, 5 are most susceptible. Cells in which an ‘h’ is listed is indication of a heterogeneous mixture. Further details on the origin of the lines in the ADP can be found in the literature [27]. (DOCX) [file pone.0156391.s003.docx]

| Variety | | Anthracnose Race | | | | | | | |
| --- | --- | --- | --- | --- | --- | --- | --- | --- | --- |
| ID | Genotype | 7 | 39 | 55 | 65 | 73 | 109 | 2047 | 3481 |
| ADP0001 | ROZIKOKO | 1.8^a^ | 4.7 | 5.0 | 0.0 | 0.2 | 3.8 | 5.0 | 0.0 |
| ADP0002 | W616444 | 1.0 | 5.0 | 5.0 | 0.0 | 0.2 | 3.5 | 5.0 | 0.3 |
| ADP0003 | KIDUNGU | 5.0 | 5.0 | 5.0 | 0.0 | 0.4 | 1.0 | 5.0 | 0.5 |
| ADP0004 | KILOMBERO | 1.5 | 5.0 | 4.5 | h | 1.0 | 2.5 | 5.0 | 1.0 |
| ADP0005 | KABUKU | 5.0 | 5.0 | 5.0 | 0.0 | 0.2 | 0.0 | 5.0 | 2.0 |
| ADP0006 | W616465 | 4.7 | 5.0 | 5.0 | 3.2 | 4.3 | 5.0 | 5.0 | 4.8 |
| ADP0007 | BUKOBA | 0.2 | 0.2 | 1.0 | 1.0 | 2.0 | 1.0 | 5.0 | 2.4 |
| ADP0008 | Nyayo | 3.0 | 4.5 | 4.8 | 0.4 | 1.3 | 0.4 | h | 0.3 |
| ADP0010 | CANADA | 1.0 | 5.0 | 5.0 | 4.6 | 2.7 | 3.0 | 5.0 | 1.5 |
| ADP0011 | KIBOROLONI | 4.7 | 5.0 | 5.0 | 0.0 | 0.0 | 0.0 | 5.0 | 1.0 |
| ADP0012 | W616489 | 4.7 | 5.0 | 5.0 | 4.5 | 4.5 | 4.8 | 5.0 | 4.2 |
| ADP0013 | KIBUMBULA | h | 5.0 | 5.0 | h | 0.5 | 1.3 | 5.0 | 0.5 |
| ADP0014 | KIANGWE | 1.0 | 3.7 | 3.0 | 4.4 | 2.2 | 3.0 | 5.0 | 1.0 |
| ADP0015 | W616495 | 5.0 | 4.8 | 5.0 | 4.4 | h | h | 5.0 | 5.0 |
| ADP0016 | GOLOLI | 4.7 | 4.2 | 4.0 | 0.0 | 0.8 | 0.0 | 5.0 | 0.3 |
| ADP0017 | W616529 | 5.0 | 4.8 | 5.0 | h | h | 5.0 | 5.0 | 5.0 |
| ADP0018 | SODAN | 5.0 | 4.7 | 5.0 | 4.8 | 4.8 | 5.0 | 5.0 | 5.0 |
| ADP0019 | KASUKANYWELE | 0.5 | 4.3 | 5.0 | 4.2 | 1.7 | 1.0 | 5.0 | 1.5 |
| ADP0020 | KIGOMA | 5.0 | 5.0 | 5.0 | 0.0 | 0.0 | 1.0 | 4.7 | 1.3 |
| ADP0021 | MBULAMTWE | 5.0 | 4.3 | 4.7 | 0.0 | 1.0 | 1.0 | 4.7 | 1.5 |
| ADP0022 | KISAPURI | 3.5 | 4.0 | 5.0 | 0.0 | 0.4 | h | 5.0 | 1.4 |
| ADP0023 | MSHORONYLONI | h | 3.3 | 5.0 | 5.0 | 4.2 | 1.8 | 5.0 | 1.5 |
| ADP0024 | YELLOW | 2.0 | 3.0 | 5.0 | h | 2.0 | 3.5 | 4.8 | 2.0 |
| ADP0025 | RUHONDELA | 4.0 | 2.6 | 5.0 | 0.0 | 0.5 | 0.0 | 5.0 | 1.0 |
| ADP0026 | Black Wonder | 2.6 | 2.2 | 5.0 | 4.2 | 2.6 | 1.0 | 4.5 | 1.2 |
| ADP0028 | Sisi | 2.7 | 1.8 | 1.2 | 4.5 | 0.7 | 0.0 | 5.0 | 2.0 |
| ADP0029 | RHNo.2 | 3.0 | 4.7 | 4.3 | 4.0 | 2.2 | h | 4.5 | 1.7 |
| ADP0030 | RHNo.6 | 0.3 | 1.0 | 2.0 | 0.2 | 1.0 | 5.0 | 5.0 | 0.3 |
| ADP0031 | RHNo.11 | 1.4 | 5.0 | 4.8 | 4.6 | h | 3.3 | 5.0 | 4.7 |
| ADP0032 | RHNo.21 | 3.0 | 1.5 | 1.7 | 3.8 | 4.3 | 4.3 | 5.0 | 4.5 |
| ADP0033 | KIJIVU | 1.3 | 5.0 | 5.0 | 0.2 | 0.2 | 1.8 | 5.0 | 0.3 |
| ADP0034 | KIJIVU | 1.7 | 4.2 | 5.0 | 0.7 | 0.0 | 3.0 | 5.0 | 0.0 |
| ADP0038 | Moono | 2.0 | 4.7 | 5.0 | 4.0 | 3.7 | 3.0 | 4.7 | 2.0 |
| ADP0039 | RoziKoko | 1.8 | 4.7 | 4.7 | 0.5 | 0.2 | 4.0 | 5.0 | 0.2 |
| ADP0041 | MRONDO | 1.3 | 3.8 | 1.7 | 3.2 | 1.0 | 1.0 | 5.0 | 0.4 |
| ADP0042 | MKOKOLA | 1.3 | 3.4 | 1.8 | 3.0 | 1.0 | 1.0 | 5.0 | 0.8 |
| ADP0044 | KIJIVU | 4.7 | 4.3 | 5.0 | 3.6 | 5.0 | 5.0 | 5.0 | 3.5 |
| ADP0045 | RHNo.12 | 1.0 | 2.3 | 5.0 | 4.2 | 3.6 | 4.0 | 3.8 | 2.5 |
| ADP0047 | MSOLINI | 1.5 | 4.8 | 5.0 | 4.2 | 3.0 | 4.0 | 5.0 | 1.2 |
| ADP0051 | RHNo.3 | 1.0 | 4.3 | 5.0 | 4.0 | 4.0 | 4.7 | 4.5 | 3.2 |
| ADP0055 | KABUKU | 5.0 | 4.5 | 5.0 | 2.2 | 3.7 | 3.3 | 2.3 | 3.0 |
| ADP0057 | KIJIVU | 4.5 | 4.8 | 5.0 | 3.6 | h | 0.0 | 5.0 | 2.2 |
| ADP0061 | Maulasi | 0.3 | 4.2 | 5.0 | 0.0 | 0.5 | 5.0 | 5.0 | 1.0 |
| ADP0064 | W616500 | 3.5 | 4.0 | 5.0 | h | h | h | 4.8 | 1.8 |
| ADP0066 | NJANO | 5.0 | 3.5 | 4.6 | 1.2 | 1.0 | 1.0 | 3.6 | 2.0 |
| ADP0071 | NJANO-DOLEA | 1.0 | 3.0 | 3.3 | 1.0 | 0.7 | 3.5 | 5.0 | 0.2 |
| ADP0072 | MASUSU | 5.0 | 3.5 | 4.8 | 4.2 | 4.0 | h | 4.2 | 3.0 |
| ADP0073 | MASUSU | 1.4 | 5.0 | 5.0 | 4.3 | 3.2 | 4.0 | 5.0 | 1.0 |
| ADP0080 | KABLANKETI | 1.0 | 2.5 | 3.8 | h | 0.4 | 1.2 | 4.3 | 3.8 |
| ADP0081 | KABLANKETI | 1.4 | 4.2 | 4.7 | 5.0 | 3.8 | 5.0 | 3.7 | 3.5 |
| ADP0089 | KABLANKETI | 0.0 | 4.4 | 4.5 | 4.8 | 3.5 | 5.0 | 3.8 | 3.2 |
| ADP0090 | KASUKANYWELE | 1.3 | 4.7 | 5.0 | h | 1.5 | 1.0 | 5.0 | 0.3 |
| ADP0092 | MORO | h | 4.8 | 4.8 | h | 4.8 | 4.0 | 4.8 | 3.0 |
| ADP0094 | LUSHALA | 1.0 | 4.5 | 4.8 | 4.8 | 4.5 | 4.0 | 4.7 | 1.3 |
| ADP0096 | Rojo | 4.5 | 0.3 | 1.0 | 4.0 | 5.0 | 5.0 | 5.0 | 4.0 |
| ADP0098 | Selian97 | 5.0 | 3.6 | 2.0 | 1.2 | 3.8 | 1.0 | 2.5 | 4.0 |
| ADP0099 | BWANASHAMBA | 5.0 | 5.0 | 5.0 | 4.7 | 5.0 | 5.0 | 5.0 | 4.5 |
| ADP0100 | EG21 | 1.3 | 5.0 | 4.3 | 0.3 | 0.7 | 5.0 | 5.0 | 0.0 |
| ADP0102 | Jesca | 1.7 | 4.7 | 5.0 | 1.0 | 0.7 | 3.7 | 5.0 | 1.5 |
| ADP0103 | Pesa | h | 4.8 | 4.5 | 1.2 | h | 3.6 | 4.8 | 1.8 |
| ADP0104 | Wiamwngu | 5.0 | 1.5 | 2.0 | 0.5 | 1.0 | 5.0 | 5.0 | 1.0 |
| ADP0105 | Sewani97 | 4.5 | 3.5 | 3.6 | 0.3 | 3.0 | 1.0 | 3.7 | 3.7 |
| ADP0106 | Zawadi | 0.6 | 2.4 | 3.3 | 0.0 | 0.0 | 5.0 | 5.0 | 1.4 |
| ADP0107 | Mishindi | 0.2 | 3.5 | 4.7 | 0.0 | 0.3 | 5.0 | 5.0 | 0.0 |
| ADP0108 | Njano | 5.0 | 4.4 | 4.6 | 0.0 | 1.7 | 1.7 | 4.6 | 0.4 |
| ADP0109 | Kablanketi | 0.3 | 4.2 | 3.7 | 3.8 | 4.3 | 3.4 | 4.8 | 2.8 |
| ADP0110 | SUG-131 | 5.0 | 4.4 | 5.0 | 0.0 | 0.0 | 0.5 | 5.0 | 0.0 |
| ADP0111 | Uyole98 | 2.2 | 0.0 | 1.6 | 1.2 | 1.2 | 1.0 | 2.0 | 0.0 |
| ADP0112 | Uyole96 | 1.0 | 2.0 | 1.2 | 0.2 | 0.3 | 3.0 | 3.0 | 0.0 |
| ADP0113 | OPS-RS4 | 5.0 | 1.5 | 1.4 | 0.4 | 0.5 | 1.0 | 3.6 | 1.0 |
| ADP0114 | OPS-RS1 | 5.0 | 2.0 | 2.0 | 0.3 | 0.5 | 1.0 | 5.0 | 2.0 |
| ADP0116 | A-800 | 0.5 | 0.7 | 1.8 | 1.5 | 0.6 | 0.0 | 5.0 | 4.2 |
| ADP0117 | A483 | 4.8 | 2.8 | 1.2 | 0.0 | 0.0 | 1.0 | 5.0 | 1.0 |
| ADP0118 | Werna | 5.0 | 3.0 | 2.0 | 0.0 | 0.0 | 1.0 | 3.6 | 0.5 |
| ADP0119 | A193 | 5.0 | 4.3 | 4.3 | 0.0 | 0.0 | 0.0 | 1.7 | 0.0 |
| ADP0121 | KranskopHR-1 | 5.0 | 1.0 | 1.0 | 0.2 | 1.0 | 0.0 | 4.8 | 0.3 |
| ADP0123 | Jenny | 5.0 | 2.0 | 2.0 | 0.6 | 1.0 | 1.0 | 5.0 | 0.5 |
| ADP0124 | Pesa | 5.0 | 4.2 | 2.0 | 2.0 | 4.5 | 4.0 | 4.8 | 4.2 |
| ADP0186 | Kisola | 1.0 | 5.0 | 4.0 | 0.5 | 0.2 | 0.0 | 5.0 | 5.0 |
| ADP0188 | G1375 | 1.2 | 5.0 | 3.2 | 0.3 | 0.3 | 4.0 | 5.0 | 0.3 |
| ADP0205 | Diacol Calima, Lyamungu 90 | 4.0 | 4.3 | 5.0 | 0.2 | 0.2 | 0.0 | 2.3 | 0.0 |
| ADP0206 | Perry Marrow | 1.8 | 5.0 | 5.0 | 0.0 | 0.7 | 5.0 | 4.6 | 0.5 |
| ADP0207 | Boca De Angel | 2.0 | 5.0 | 5.0 | 0.0 | 0.0 | 4.0 | 4.8 | 0.0 |
| ADP0208 | Limoncillo | 1.7 | 5.0 | 4.2 | 0.2 | 0.0 | 2.3 | 3.0 | 0.0 |
| ADP0211 | G 4780 | 2.8 | 1.0 | 4.7 | 0.0 | 0.0 | 0.0 | 1.2 | 0.4 |
| ADP0212 | Gordinho | 3.0 | 5.0 | 5.0 | 0.0 | 0.2 | 1.0 | 3.0 | 5.0 |
| ADP0213 | Manteigao, Roxo Gigante | 5.0 | 5.0 | 4.8 | 0.0 | 0.0 | 1.2 | 5.0 | 4.3 |
| ADP0214 | Manteigao Preto | 4.2 | 4.5 | 3.7 | 0.0 | 0.0 | 0.0 | 4.8 | 1.0 |
| ADP0220 | G5625 | 4.2 | 4.3 | 4.8 | 0.0 | 0.3 | 5.0 | 5.0 | 0.5 |
| ADP0224 | Yellow Eye | 3.7 | 3.7 | 3.0 | 0.7 | 0.4 | 5.0 | 5.0 | 1.5 |
| ADP0225 | Mecosta 003 | 3.5 | 1.8 | 0.5 | 0.7 | 1.0 | 4.5 | 5.0 | 1.0 |
| ADP0232 | G 7930 | 1.0 | 4.4 | 2.0 | 0.2 | 0.0 | 3.5 | 5.0 | 1.2 |
| ADP0242 | Tailor Horticultural | 4.8 | 3.7 | 4.5 | 4.4 | 4.7 | 4.7 | 5.0 | 5.0 |
| ADP0269 | Horoz Fasulyesi | 5.0 | 2.5 | 4.0 | 0.0 | 0.7 | 5.0 | 5.0 | 0.7 |
| ADP0277 | G13778 | 1.0 | 4.5 | 5.0 | 0.0 | 0.3 | 3.0 | 4.8 | 1.5 |
| ADP0280 | G14440 | 5.0 | 5.0 | 5.0 | 4.7 | 5.0 | 4.7 | 5.0 | 5.0 |
| ADP0303 | G17913 | 1.0 | 4.4 | 4.8 | 0.0 | 0.5 | 3.0 | 5.0 | 1.0 |
| ADP0310 | Frutilla Canete | 5.0 | 5.0 | 5.0 | 0.2 | 0.3 | h | 5.0 | h |
| ADP0351 | Georgia 1025/1983 | 5.0 | 4.8 | 5.0 | 5.0 | 5.0 | 5.0 | 5.0 | 5.0 |
| ADP0353 | Masusu | 1.0 | 5.0 | 5.0 | 4.7 | 3.8 | 3.6 | 5.0 | 1.0 |
| ADP0354 | G22502 | 4.3 | 2.5 | 5.0 | 0.3 | 0.6 | 1.0 | 2.0 | 1.0 |
| ADP0366 | Line 235 | 1.0 | 4.7 | 5.0 | 1.0 | 0.2 | 2.0 | 5.0 | 0.7 |
| ADP0368 | Line 258 | 0.6 | 4.5 | 3.3 | 0.4 | 0.0 | 1.0 | 4.8 | 1.0 |
| ADP0376 | PI189408 | 2.2 | 4.6 | 5.0 | 0.3 | 0.2 | 1.5 | 4.4 | 0.5 |
| ADP0390 | PI307808 | 5.0 | 4.7 | 5.0 | 0.2 | 0.0 | 0.0 | 4.3 | 0.2 |
| ADP0391 | PI308894 | 5.0 | 3.0 | 5.0 | 0.0 | 0.0 | 0.0 | 3.7 | 0.0 |
| ADP0392 | PI309701 | 4.8 | 1.6 | 5.0 | h | h | 4.0 | 4.0 | h |
| ADP0395 | PI310511 | 4.0 | 0.6 | 5.0 | 0.0 | 0.5 | 0.0 | 2.2 | 0.3 |
| ADP0417 | PI451906 | 1.2 | 1.6 | 5.0 | 4.8 | 4.7 | 3.5 | 5.0 | 0.6 |
| ADP0427 | Badillo | 4.0 | 0.0 | 1.0 | 2.6 | 5.0 | 4.4 | 4.8 | 5.0 |
| ADP0428 | Colorado del Pais | 4.0 | 0.0 | 1.3 | h | h | 0.0 | 5.0 | 5.0 |
| ADP0429 | PR9920-171 | 5.0 | 4.0 | 5.0 | 4.8 | 5.0 | 0.0 | 4.5 | 4.3 |
| ADP0430 | PR1013-3 | 5.0 | 2.2 | 2.7 | 4.6 | 5.0 | 5.0 | 4.6 | 4.5 |
| ADP0431 | Gurabo5 | 1.2 | 3.0 | 1.3 | 5.0 | 5.0 | 5.0 | 5.0 | 4.8 |
| ADP0432 | PR0637-134 | 3.6 | 1.0 | 1.0 | 0.3 | 0.5 | 1.0 | 1.3 | 1.0 |
| ADP0433 | PR9745-232 | 5.0 | 2.0 | 1.0 | 3.8 | 5.0 | 5.0 | 5.0 | 5.0 |
| ADP0435 | RM-05-07 | 5.0 | 2.5 | 2.0 | 1.0 | 1.0 | 1.0 | 1.5 | 2.5 |
| ADP0437 | PC-50 | 1.8 | 5.0 | 5.0 | 1.0 | 1.0 | 5.0 | 5.0 | 0.8 |
| ADP0438 | 46-1 | 0.5 | 4.7 | 1.5 | 5.0 | 4.3 | 5.0 | 5.0 | 1.0 |
| ADP0442 | LargaComercial | 1.3 | 4.8 | 5.0 | 0.0 | 0.2 | 3.2 | 4.2 | 0.0 |
| ADP0450 | INIAP422 | 5.0 | 4.8 | 2.0 | 1.0 | 1.3 | 1.0 | 4.2 | 5.0 |
| ADP0459 | PI331356-C | 5.0 | 5.0 | 5.0 | 0.0 | 0.2 | 1.0 | 4.7 | 0.0 |
| ADP0460 | PI331356-B | 1.7 | 5.0 | 5.0 | 0.3 | 0.5 | 4.0 | 5.0 | 0.8 |
| ADP0463 | PI353534-A | 2.0 | 0.7 | 1.4 | 0.0 | 0.4 | 2.6 | 4.7 | 0.3 |
| ADP0466 | PI449430 | 1.2 | 4.8 | 4.7 | 0.4 | 0.2 | 2.0 | 4.7 | 0.0 |
| ADP0467 | PI209808 | 1.3 | 4.0 | 5.0 | 1.0 | 0.0 | 3.5 | 4.8 | 0.3 |
| ADP0468 | N/A | 1.2 | 4.7 | 5.0 | 0.5 | 0.4 | 3.0 | 4.7 | 0.2 |
| ADP0469 | PI527521 | 2.0 | h | 5.0 | 0.5 | 1.0 | 5.0 | 4.8 | 0.0 |
| ADP0470 | PI527508 | h | 4.2 | 5.0 | h | 5.0 | 4.7 | 4.8 | 4.5 |
| ADP0474 | PI527519 | 0.7 | 5.0 | 5.0 | 1.0 | 0.7 | 2.4 | 4.7 | 0.0 |
| ADP0475 | PI319706 | h | 4.8 | 4.8 | 3.0 | 3.0 | 3.0 | 4.7 | 1.2 |
| ADP0476 | Hutterite | 4.8 | 4.7 | 1.2 | 4.0 | 5.0 | 5.0 | 4.5 | 2.6 |
| ADP0477 | PI527512 | 4.8 | 3.8 | 5.0 | 1.3 | 0.4 | 1.8 | 3.6 | 0.3 |
| ADP0479 | PI527530 | 2.7 | 1.5 | 1.7 | 4.8 | 1.0 | 1.0 | 5.0 | 1.6 |
| ADP0480 | PI209804 | 1.2 | 5.0 | 5.0 | 0.0 | 0.0 | 4.0 | 5.0 | 0.0 |
| ADP0481 | PI449428 | 3.0 | 4.8 | 5.0 | 0.0 | 0.3 | 4.0 | 5.0 | 1.2 |
| ADP0482 | PI209802 | 1.7 | 5.0 | 5.0 | 0.0 | 0.5 | 3.3 | 5.0 | 0.2 |
| ADP0483 | PI209815 | 5.0 | 4.8 | 5.0 | 0.0 | h | 4.7 | 4.0 | h |
| ADP0509 | Fernando | 1.4 | 4.5 | 4.5 | 2.8 | 3.0 | 1.7 | 4.2 | 2.0 |
| ADP0512 | Ervilha | h | 4.7 | 4.0 | h | 2.8 | 0.6 | 5.0 | 5.0 |
| ADP0518 | Mantegablanca,  Kibala | 0.0 | 4.0 | 4.5 | 3.8 | 4.5 | 2.0 | 5.0 | 1.2 |
| ADP0519 | Katarina,Cela | 0.0 | 5.0 | 4.5 | 0.6 | 0.4 | 0.0 | 4.7 | 1.4 |
| ADP0520 | Chumbo,Cela | 0.3 | 4.7 | 5.0 | h | h | 5.0 | 5.0 | 5.0 |
| ADP0522 | Amarelo,Cela | 0.5 | 1.0 | 1.7 | 1.5 | 4.2 | 3.3 | 3.0 | 2.0 |
| ADP0523 | Canario,Cela | 0.2 | 5.0 | 5.0 | 0.8 | 0.3 | 0.0 | 5.0 | 5.0 |
| ADP0598 | Charlevoix | 2.0 | 5.0 | 5.0 | 0.2 | 0.2 | 4.2 | 5.0 | 0.3 |
| ADP0599 | Isles | 0.0 | 0.0 | 1.4 | 0.4 | 0.0 | 2.0 | 5.0 | 1.8 |
| ADP0600 | K07921 | 5.0 | 0.6 | 1.0 | 1.5 | 0.0 | 4.5 | 5.0 | 1.0 |
| ADP0601 | Camelot | 4.6 | 1.7 | 1.0 | 0.3 | 0.0 | 5.0 | 4.8 | 0.0 |
| ADP0602 | Sacramento | 5.0 | 3.3 | h | 1.0 | 1.0 | 4.0 | 5.0 | 1.4 |
| ADP0603 | Wallace773-V98 | 5.0 | 1.6 | 1.5 | 5.0 | 5.0 | 5.0 | 5.0 | 5.0 |
| ADP0604 | 1062-V98 | 5.0 | 2.0 | 2.0 | 0.5 | 0.6 | 5.0 | 5.0 | 0.0 |
| ADP0605 | 1132-V96 | 5.0 | 0.0 | 1.0 | h | h | 4.0 | 4.8 | 4.8 |
| ADP0606 | NY104 | 4.3 | 1.5 | 1.0 | 0.5 | 0.0 | 3.7 | 4.5 | 0.5 |
| ADP0607 | NY105 | 4.0 | 1.5 | 1.5 | 0.2 | 0.0 | 4.0 | 5.0 | 0.4 |
| ADP0608 | UI-51 | 5.0 | 0.0 | 2.0 | 5.0 | 5.0 | 5.0 | 4.8 | 5.0 |
| ADP0609 | K-407 | 5.0 | 0.7 | 1.5 | 0.5 | 1.0 | 0.0 | 5.0 | 0.3 |
| ADP0610 | G-122 | 5.0 | 5.0 | 5.0 | 0.0 | 0.0 | 0.0 | 5.0 | 0.2 |
| ADP0611 | PompadourB | 1.2 | 5.0 | 5.0 | 4.7 | 5.0 | 5.0 | 5.0 | 1.7 |
| ADP0612 | ICAQuimbaya | 2.8 | 5.0 | 5.0 | 1.0 | 0.3 | 3.5 | 5.0 | 0.8 |
| ADP0613 | 02-385-14 | 1.0 | 1.6 | 1.5 | 1.0 | 0.5 | 4.5 | 4.6 | 0.3 |
| ADP0614 | ND061106 | 4.2 | 1.4 | 2.0 | 0.0 | 0.2 | 5.0 | 5.0 | 0.2 |
| ADP0615 | Litekid | 1.2 | 4.8 | 5.0 | 1.0 | 0.3 | 5.0 | 5.0 | 0.2 |
| ADP0616 | OACLyrick | 4.8 | 1.8 | 1.6 | 1.0 | 1.0 | 4.5 | 4.8 | 0.2 |
| ADP0617 | RedRider | 5.0 | 4.2 | 5.0 | h | h | 5.0 | 5.0 | 5.0 |
| ADP0618 | ACElk | 4.2 | 2.3 | 0.8 | 1.2 | 0.3 | 4.3 | 4.5 | 0.0 |
| ADP0619 | UCD0906 | 4.8 | 1.0 | 1.2 | 3.5 | 5.0 | 5.0 | 5.0 | 4.7 |
| ADP0620 | UCD0405 | 4.6 | 4.5 | 5.0 | 4.0 | 4.3 | 5.0 | 5.0 | 4.7 |
| ADP0621 | JaloEEP558 | 4.6 | 5.0 | 5.0 | 0.3 | 0.2 | 0.0 | 5.0 | 1.0 |
| ADP0622 | UCD0701 | 5.0 | 1.7 | 1.5 | 3.2 | 5.0 | 5.0 | 5.0 | 4.8 |
| ADP0623 | Drake | 1.2 | 1.3 | 2.0 | 0.2 | 0.2 | 5.0 | 5.0 | 0.7 |
| ADP0624 | Dolly | 5.0 | 2.0 | 1.8 | 0.0 | 0.2 | 5.0 | 4.8 | 0.5 |
| ADP0625 | Micran | 4.8 | 4.0 | 5.0 | 0.0 | 0.0 | 5.0 | 4.0 | 0.5 |
| ADP0627 | H9659-21-1 | 1.0 | 4.3 | 5.0 | 3.0 | 4.4 | 3.0 | 5.0 | 0.3 |
| ADP0628 | H9659-27-7 | 1.5 | 0.3 | 1.3 | 0.0 | 0.3 | 3.0 | 5.0 | 0.2 |
| ADP0629 | H9659-27-10 | 1.2 | 1.7 | 1.5 | 3.5 | 3.4 | 2.6 | 5.0 | 0.0 |
| ADP0630 | H9659-23-1 | 1.0 | h | 5.0 | 5.0 | 4.4 | 3.7 | 5.0 | 0.3 |
| ADP0631 | OAC Inferno | 1.5 | 1.5 | 1.0 | 0.0 | 0.3 | 4.7 | 5.0 | 0.0 |
| ADP0632 | TARSHT1 | 5.0 | 1.0 | 1.0 | 4.7 | 5.0 | 5.0 | 4.8 | 4.2 |
| ADP0633 | TARS-HT2 | 5.0 | 5.0 | 5.0 | 4.5 | 5.0 | 5.0 | 4.8 | 4.7 |
| ADP0634 | UC Red Kidney | 5.0 | 5.0 | 5.0 | 0.0 | 0.2 | 4.0 | 5.0 | 1.3 |
| ADP0635 | OAC Redstar | 1.7 | 2.0 | 2.0 | 0.0 | 0.4 | 5.0 | 5.0 | 0.2 |
| ADP0636 | Montcalm | 4.2 | 2.0 | 1.8 | 0.0 | 0.2 | 5.0 | 5.0 | 0.2 |
| ADP0637 | Isabella | 5.0 | 2.3 | 2.0 | 3.0 | 3.0 | 4.0 | 4.8 | 4.3 |
| ADP0638 | Red Hawk | 1.3 | 0.5 | 1.5 | 0.0 | 1.3 | 3.3 | 5.0 | 0.7 |
| ADP0639 | Chinook2000 | 0.3 | 0.3 | 1.0 | 0.0 | 1.2 | 5.0 | 5.0 | 0.2 |
| ADP0640 | Beluga | 5.0 | 2.0 | 1.0 | 0.4 | 0.5 | 4.3 | 4.7 | 0.2 |
| ADP0642 | Taylor Hort. | 5.0 | 0.0 | 2.0 | 4.5 | 5.0 | 5.0 | 4.8 | 5.0 |
| ADP0643 | Cardinal | 5.0 | 2.0 | 0.6 | 4.0 | 5.0 | 5.0 | 5.0 | 4.7 |
| ADP0644 | Fox Fire | 5.0 | 2.0 | 1.5 | 4.3 | 5.0 | 5.0 | 5.0 | 4.4 |
| ADP0646 | Myasi | 5.0 | 5.0 | 5.0 | 4.8 | 5.0 | 5.0 | 5.0 | 4.7 |
| ADP0647 | Red Kanner | 3.0 | 1.2 | 1.0 | 3.8 | 4.5 | 4.0 | 5.0 | 4.0 |
| ADP0648 | Red Kloud | 5.0 | 1.6 | 1.7 | 4.0 | 4.8 | 5.0 | 5.0 | 4.0 |
| ADP0649 | Kamiakin | 4.8 | 1.7 | 2.0 | 0.0 | 1.0 | 0.0 | 5.0 | 0.0 |
| ADP0650 | K-42 | 5.0 | 2.0 | 1.8 | 0.0 | 1.0 | 0.0 | 4.5 | 0.0 |
| ADP0651 | K-59 | 4.0 | 1.5 | 1.0 | 0.0 | 0.5 | 5.0 | 5.0 | 1.0 |
| ADP0652 | Lisa | 5.0 | 4.8 | 5.0 | 0.3 | 1.2 | 5.0 | 5.0 | 1.5 |
| ADP0653 | USDK-CBB-15 | 0.0 | 0.2 | 0.5 | 0.0 | 0.0 | 3.3 | 5.0 | 0.0 |
| ADP0654 | USDK-4 | 3.5 | 0.5 | 1.0 | 0.3 | 0.0 | 5.0 | 5.0 | 1.5 |
| ADP0655 | Fiero | 3.8 | 1.0 | 1.5 | 1.0 | 0.0 | 5.0 | 5.0 | 1.0 |
| ADP0656 | Royal Red | 5.0 | 1.7 | 1.0 | 0.2 | 0.2 | 0.0 | 5.0 | 0.8 |
| ADP0657 | Kardinal | 4.3 | 2.0 | 1.5 | 0.2 | 0.2 | 0.0 | 4.8 | 0.5 |
| ADP0658 | Blush | 4.5 | 0.0 | 0.8 | 0.0 | 0.0 | 0.0 | 4.7 | 0.2 |
| ADP0659 | USLK-1 | 4.0 | 0.7 | 1.5 | 1.0 | 1.0 | 4.0 | 4.2 | 0.8 |
| ADP0660 | Krimson | 1.0 | 0.3 | 1.0 | 1.3 | 0.8 | 3.7 | 4.8 | 0.0 |
| ADP0661 | USCR-7 | 5.0 | 3.0 | 3.3 | 1.2 | 1.7 | 0.3 | 5.0 | 5.0 |
| ADP0662 | USCR-9 | 5.0 | 2.0 | 1.7 | 4.3 | 5.0 | 5.0 | 5.0 | 5.0 |
| ADP0663 | USCR-CBB-20 | 1.2 | 1.7 | 1.0 | 0.0 | 0.0 | 3.3 | 5.0 | 0.5 |
| ADP0664 | Silver Cloud | 5.0 | 2.0 | 1.0 | 0.5 | 0.5 | 1.0 | 4.7 | 2.0 |
| ADP0665 | USWK-CBB-17 | 4.0 | 0.5 | 1.0 | 3.6 | 4.5 | 4.0 | 5.0 | 5.0 |
| ADP0666 | USWK-6 | 4.7 | 1.0 | 1.5 | 0.3 | 0.7 | 3.8 | 5.0 | 0.4 |
| ADP0667 | VA-19 | 4.0 | 1.8 | 1.0 | 0.0 | 1.0 | 3.2 | 5.0 | 0.3 |
| ADP0670 | AC Calmont | 3.8 | 1.0 | 1.4 | 0.2 | 0.0 | 3.4 | 5.0 | 1.0 |
| ADP0672 | CDRK | 5.0 | 5.0 | 5.0 | 1.0 | 0.0 | 0.0 | 5.0 | 0.2 |
| ADP0673 | UC Nichols | 5.0 | 1.5 | 1.7 | 0.2 | 1.0 | 5.0 | 5.0 | 0.0 |
| ADP0674 | UCD0704 | 2.0 | 1.0 | 1.0 | 0.4 | 1.0 | 1.0 | 3.7 | 0.5 |
| ADP0675 | UCD0801 | 5.0 | 2.0 | 2.0 | 3.5 | 5.0 | 5.0 | 5.0 | 5.0 |
| ADP0676 | CELRK | 4.0 | 2.0 | 2.0 | 0.3 | 1.0 | 4.0 | 5.0 | 0.3 |
| ADP0677 | Etna | 4.8 | 1.7 | 2.0 | 3.4 | 5.0 | 5.0 | 5.0 | 5.0 |
| ADP0678 | Hooter | 4.2 | 2.0 | 1.5 | 0.0 | 0.0 | 1.0 | 3.3 | 0.2 |
| ADP0679 | Red Rover | 4.2 | 1.2 | 1.7 | 0.0 | 0.0 | 5.0 | 5.0 | 0.3 |
| ADP0680 | Clouseau | 4.3 | 2.0 | 3.5 | 0.0 | 1.0 | 4.0 | 4.8 | 0.0 |
| ADP0683 | IJR | 4.8 | 5.0 | 5.0 | 2.7 | 5.0 | 4.3 | 5.0 | 5.0 |
| ADP0684 | Majesty | 5.0 | 3.0 | 2.7 | 0.5 | 1.2 | 5.0 | 5.0 | 1.0 |
| ADP0686 | ADP0686 | 5.0 | 2.5 | 1.6 | 1.0 | 1.0 | 4.5 | 5.0 | 1.3 |
| ADP0687 | Pink Panther | 4.5 | 2.2 | 2.0 | 1.0 | 1.2 | 4.0 | 5.0 | 1.2 |
