# Supplementary material for: Genome-Wide Association Study of Anthracnose Resistance in Andean Beans (Phaseolus vulgaris)
Source: PLoS One. 2016 Jun 6;11(6):e0156391. doi: 10.1371/journal.pone.0156391 (PMC4894742; doi:10.1371/journal.pone.0156391)
Supplement: S2 Table — aDisease scores are mean of six plants, 0 are most resistant, 5 are most susceptible [30]. (DOCX) [file pone.0156391.s004.docx]

| Variety | | Anthracnose Race | | | | | | | |
| --- | --- | --- | --- | --- | --- | --- | --- | --- | --- |
| ID | Genotype | 7 | 39 | 55 | 65 | 73 | 109 | 2047 | 3481 |
| ADP0007 | Bukoba | 0.2^a^ | 0.2 | 1.0 | 1.0 | 2.0 | 1.0 | 5.0 | 2.4 |
| ADP0030 | RHNo.6 | 0.3 | 1.0 | 2.0 | 0.2 | 1.0 | 5.0 | 5.0 | 0.3 |
| ADP0111 | Uyole98 | 2.2 | 0.0 | 1.6 | 1.2 | 1.2 | 1.0 | 2.0 | 0.0 |
| ADP0112 | Uyole96 | 1.0 | 2.0 | 1.2 | 0.2 | 0.3 | 3.0 | 3.0 | 0.0 |
| ADP0113 | OPS-RS4 | 5.0 | 1.5 | 1.4 | 0.4 | 0.5 | 1.0 | 3.6 | 1.0 |
| ADP0116 | A-800 | 0.5 | 0.7 | 1.8 | 1.5 | 0.6 | 0.0 | 5.0 | 4.2 |
| ADP0121 | Kranskop HR-1 | 5.0 | 1.0 | 1.0 | 0.2 | 1.0 | 0.0 | 4.8 | 0.3 |
| ADP0211 | G 4780 | 2.8 | 1.0 | 4.7 | 0.0 | 0.0 | 0.0 | 1.2 | 0.4 |
| ADP0432 | PR0637-134 | 3.6 | 1.0 | 1.0 | 0.3 | 0.5 | 1.0 | 1.3 | 1.0 |
| ADP0463 | PI353534-A | 2.0 | 0.7 | 1.4 | 0.0 | 0.4 | 2.6 | 4.7 | 0.3 |
| ADP0599 | Isles | 0.0 | 0.0 | 1.4 | 0.4 | 0.0 | 2.0 | 5.0 | 1.8 |
| ADP0609 | K-407 | 5.0 | 0.7 | 1.5 | 0.5 | 1.0 | 0.0 | 5.0 | 0.3 |
| ADP0613 | 02-385-14 | 1.0 | 1.6 | 1.5 | 1.0 | 0.5 | 4.5 | 4.6 | 0.3 |
| ADP0623 | Drake | 1.2 | 1.3 | 2.0 | 0.2 | 0.2 | 5.0 | 5.0 | 0.7 |
| ADP0628 | H9659-27-7 | 1.5 | 0.3 | 1.3 | 0.0 | 0.3 | 3.0 | 5.0 | 0.2 |
| ADP0631 | OAC Inferno | 1.5 | 1.5 | 1.0 | 0.0 | 0.3 | 4.7 | 5.0 | 0.0 |
| ADP0638 | Red Hawk | 1.3 | 0.5 | 1.5 | 0.0 | 1.3 | 3.3 | 5.0 | 0.7 |
| ADP0639 | Chinook 2000 | 0.3 | 0.3 | 1.0 | 0.0 | 1.2 | 5.0 | 5.0 | 0.2 |
| ADP0650 | K-42 | 5.0 | 2.0 | 1.8 | 0.0 | 1.0 | 0.0 | 4.5 | 0.0 |
| ADP0653 | USDK-CBB-15 | 0.0 | 0.2 | 0.5 | 0.0 | 0.0 | 3.3 | 5.0 | 0.0 |
| ADP0656 | Royal Red | 5.0 | 1.7 | 1.0 | 0.2 | 0.2 | 0.0 | 5.0 | 0.8 |
| ADP0657 | Kardinal | 4.3 | 2.0 | 1.5 | 0.2 | 0.2 | 0.0 | 4.8 | 0.5 |
| ADP0658 | Blush | 4.5 | 0.0 | 0.8 | 0.0 | 0.0 | 0.0 | 4.7 | 0.2 |
| ADP0660 | Krimson | 1.0 | 0.3 | 1.0 | 1.3 | 0.8 | 3.7 | 4.8 | 0.0 |
| ADP0663 | USCR-CBB-20 | 1.2 | 1.7 | 1.0 | 0.0 | 0.0 | 3.3 | 5.0 | 0.5 |
| ADP0664 | Silver Cloud | 5.0 | 2.0 | 1.0 | 0.5 | 0.5 | 1.0 | 4.7 | 2.0 |
| ADP0674 | UCD0704 | 2.0 | 1.0 | 1.0 | 0.4 | 1.0 | 1.0 | 3.7 | 0.5 |
| ADP0678 | Hooter | 4.2 | 2.0 | 1.5 | 0.0 | 0.0 | 1.0 | 3.3 | 0.2 |
