# Supplementary material for: Genome-Wide Association Study of Anthracnose Resistance in Andean Beans (Phaseolus vulgaris)
Source: PLoS One. 2016 Jun 6;11(6):e0156391. doi: 10.1371/journal.pone.0156391 (PMC4894742; doi:10.1371/journal.pone.0156391)
Supplement: S3 Table — aA, Andean, MA, Mesoamerican or Mx Mixed gene pool designation of the race is based on level of virulence on host differentials from either gene pool; Further information on races is provided in the literature [6, 28]. (DOCX) [file pone.0156391.s005.docx]

| Race | Gene Pool^a^ | Resistant Lines | Susceptible Lines | Heterogeneous Lines | % resistant |
| --- | --- | --- | --- | --- | --- |
| 7 | A | 85 | 134 | 7 | 37.6 |
| 39 | A | 94 | 130 | 2 | 41.6 |
| 55 | A | 93 | 132 | 1 | 41.2 |
| 65 | MA | 143 | 68 | 15 | 63.3 |
| 73 | MA | 148 | 66 | 12 | 65.5 |
| 109 | A | 78 | 142 | 6 | 34.5 |
| 2047 | Mx | 10 | 215 | 1 | 4.4 |
| 3481 | Mx | 161 | 62 | 3 | 71.2 |
