# Supplementary material for: Genome-Wide Association Study of Anthracnose Resistance in Andean Beans (Phaseolus vulgaris)
Source: PLoS One. 2016 Jun 6;11(6):e0156391. doi: 10.1371/journal.pone.0156391 (PMC4894742; doi:10.1371/journal.pone.0156391)
Supplement: S4 Table — **significant at Pr(F) = 0.01%; further information on SNP can be found in literature [32]. (DOCX) [file pone.0156391.s006.docx]

| SNP marker | SNP position (Mb) | Pr (F)** | R^2^ |
| --- | --- | --- | --- |
| ss715649883 | 37.652001 | 0.000000002 | 0.3202 |
| ss715645856 | 49.617274 | 0.000000055 | 0.2726 |
| ss715645287 | 49.793139 | 0.000000000 | 0.5828 |
| ss715645285 | 49.853879 | 0.000000000 | 0.6447 |
| ss715645284 | 49.862290 | 0.000000000 | 0.5986 |
| ss715645280 | 49.895862 | 0.000000000 | 0.6709 |
| ss715645275 | 49.953420 | 0.000000000 | 0.6333 |
| ss715645270 | 50.014600 | 0.000000000 | 0.6397 |
| ss715645262 | 50.099818 | 0.000000000 | 0.6112 |
| ss715645259 | 50.130201 | 0.000000000 | 0.6714 |
| ss715645258 | 50.155987 | 0.000000000 | 0.6935 |
| ss715645252 | 50.222584 | 0.000000000 | 0.6531 |
| ss715645251 | 50.301592 | 0.000000000 | 0.6779 |
